# Intra- and Inter-Brain Synchronization during Musical Improvisation on the Guitar

**DOI:** 10.1371/journal.pone.0073852

**Published:** 2013-09-10

**Authors:** Viktor Müller, Johanna Sänger, Ulman Lindenberger

**Affiliations:** Center for Lifespan Psychology, Max Planck Institute for Human Development, Berlin, Germany; Indiana University, United States of America

## Abstract

Humans interact with the environment through sensory and motor acts. Some of these interactions require synchronization among two or more individuals. Multiple-trial designs, which we have used in past work to study interbrain synchronization in the course of joint action, constrain the range of observable interactions. To overcome the limitations of multiple-trial designs, we conducted single-trial analyses of electroencephalography (EEG) signals recorded from eight pairs of guitarists engaged in musical improvisation. We identified hyper-brain networks based on a complex interplay of different frequencies. The intra-brain connections primarily involved higher frequencies (e.g., beta), whereas inter-brain connections primarily operated at lower frequencies (e.g., delta and theta). The topology of hyper-brain networks was frequency-dependent, with a tendency to become more regular at higher frequencies. We also found hyper-brain modules that included nodes (i.e., EEG electrodes) from both brains. Some of the observed network properties were related to musical roles during improvisation. Our findings replicate and extend earlier work and point to mechanisms that enable individuals to engage in temporally coordinated joint action.

## Introduction

In daily life, people must often coordinate their actions with those of others. Recent research indicates that synchronized brain activity accompanies coordinated behavior [1]–[6]. Oscillatory couplings also have been observed for other biological functions, such as respiration and cardiac activity during choir singing [7]. However, the neural mechanisms that implement interpersonally coordinated behavior remain elusive [8]–[10].

In a previous study [4], we observed duets of guitarists playing a short melody in unison. We found that phase synchronization within and between brains increased significantly during periods of preparatory metronome tempo setting and coordinated play onset. In addition, phase alignment extracted from within-brain dynamics was related to behavioral play onset asynchrony between guitarists. Using a similar paradigm, Sänger et al. [6] found that phase locking as well as within-brain and between-brain phase-coherence connection strengths were enhanced at frontal and central electrodes during periods that put particularly high demands on musical coordination. Phase locking was modulated in relation to the experimentally assigned musical roles of leader and follower, corroborating the functional significance of synchronous oscillations in dyadic music performance. In addition, graph theory analyses revealed within-brain and hyper-brain networks with small-world properties that were enhanced during musical coordination periods as well as community structures encompassing electrodes from both brains, termed *hyper-brain modules*. Taken together, these findings suggest that between-brain synchronization during duet playing cannot be reduced to processing similarities induced by attending to similar or identical external stimuli. Instead, between-brain synchronization also reflects, to some extent at least, the behavioral dynamics between the two players.

Related work from other groups supports the view that interbrain synchronization is functionally related to interpersonal action coordination. Dumas and colleagues [3] investigated the spontaneous imitation of hand movements recorded with a dual-video and dual-EEG setup. Using video recordings, they classified segments of behavior as synchronous or non-synchronous. When comparing the two, they found statistically significant phase locking across time between electrodes of the model and the imitator, predominantly in episodes of synchrony among alpha-mu, beta and gamma frequency bands. Others have investigated directed coupling between different electrodes or cortical regions both within and between brains [1], [2], [11], [12] with measures based on the concept of Granger Causality [13]. In this context, measures derived from graph theory, which is increasingly popular in the neurosciences in general [14]–[21], have also been successfully applied in inter-brain studies [1], [2], [6]. The functional role of the different frequency bands combined with a graph-theoretical approach (GTA) has been taken into account in these studies (e.g., [1], [2], [6]), however, the differential contributions of different frequencies to intra- and inter-brain couplings have remained unclear. Furthermore, frequency-specific properties of hyper-brain network topology during interpersonal action coordination or musical improvisation have not been studied thus far.

In this study, we aim to describe and quantify the individual and joint brain networks of guitarists improvising jazz together in pairs. For this purpose, we applied new coupling measures based on the phase dynamics of synchronous brain oscillations within and between the brains, which we then fed into a graph-theoretical evaluation of “hyper-brain networks” [2], [6]. In doing so, we wished to investigate three sets of key issues.

First, we examined the general properties of brain networks during an activity that is likely to involve all of the following: (a) many degrees of freedom (e.g., improvisation instead of playing from a score), (b) a strong emphasis on interpersonal action coordination, and (c) a strong need to engage in mentalizing (e.g., trying to imagine what the musical partner will do next). We assumed that an activity marked by these properties would give rise to a *hyper-brain module* that is defined by coordinated activity within and between the two brains.

Second, we were interested in exploring differential contributions of within-brain and between-brain partitions of the network to the hyper-brain network as a whole. For this purpose, we analyzed the intra- and inter-brain connectivity for 17 frequency components or frequencies of interest (FOI) between 2 and 28 Hz. Based on findings in the literature that are related to coordinated behavior [4], [6], [22], [23], we decided to restrict our analyses to frequency bands below 30 Hz. This decision was further motivated by evidence indicating that low-frequency EEG or MEG (magnetoencephalography) oscillations (up to 30 Hz) are involved in limb and hand movement control [24]–[28] and in sensorimotor integration [29], which are essential during guitar playing. For this reason, we expected that inter-brain synchronization at higher gamma frequency, which has been observed in a few studies [1], [3], would be of minor importance. We expected that intra- and inter-brain coupling would involve different oscillatory networks, either preferring high (e.g., within brains) or low (e.g., between brains) communication frequencies. This expectation was based on the assumption that inter-brain communication is operating at longer timescales than intra-brain coupling.

Third, we explored whether different musical roles would map onto differences in brain network properties. We expected that the coupling strength would be stronger when the two guitarists were playing together than when one guitarist was playing and the other was listening, as the former, on average, may require more coordination. For the same reason, we expected differences in network topology in these two different situations, which would also be frequency dependent, given the assumed differential contributions of intra-brain and inter-brain portions of the network to the hyper-brain structure.

## Methods

### Participants

Nine pairs of professional guitarists participated in the study. One pair was excluded from data analysis because of recording artifacts. Analyses presented here are based on the remaining eight pairs of guitarists. One guitarist was a member of four of the pairs. Each of the remaining four pairs involved different guitarists. Thus altogether, 13 guitarists participated in the study. Participants’ mean age was 29.5 years (SD = 10.0). All participants were right-handed and had been playing the guitar professionally for more than 5 years (mean = 15.7 years, SD = 9.3). The Ethics Committee of Max Planck Institute for Human Development approved the study, and it was performed in accordance with the ethical standards laid down in the 1964 Declaration of Helsinki. All participants volunteered for this experiment and gave their written informed consent prior to their inclusion in the study. The subject of the movie (Movie S1) had given written informed consent, as outlined in the PLOS consent form, for the publication of the movie.

### EEG Data Acquisition and Preprocessing

EEG measurement took place in an acoustically and electromagnetically shielded cabin. Each pair of participants took part in three sessions of 5–7 min each. During the first session, guitarist A freely played a melody (improvisation) while guitarist B was listening (Play A). During the second session, guitarist B played and guitarist A listened (Play B). During the third session, both guitarists played a duet improvisation (Play AB). Typically, this would mean that one guitarist played a melody, while the other one was accompanying him or her with chords. Seven of the pairs switched roles several times during the improvisation; in the remaining two pairs, one of the two guitarists played the melody all the time. In contrast to our previous studies [4], [6], we did not use a metronome. Hence, guitarists were free to choose their preferred tempo.

Measurement took place continuously throughout the sessions. Through one microphone each, the sounds of the guitars were recorded on two EEG channels simultaneously with the EEG recordings. In addition, video and sound were recorded using Video Recorder Software (Brain Products, Munich, Germany), synchronized with EEG data acquisition (a video recording of a pair of guitarists playing an improvisation together, with the corresponding EEG and microphone-channel recordings can be found in the Movie S1). Microphone and video recordings were used for the offline setting of event triggers in the EEG recordings later on. EEG was simultaneously recorded from both participants using two electrode caps with 64 Ag/AgCl electrodes each, placed according to the international 10–10 system, with the reference electrode at the right mastoid. The vertical and horizontal electrooculogram (EOG) was recorded to control for eye blinks and eye movements. The sampling rate was 5000 Hz. Recorded frequency bands ranged from 0.01 to 1000 Hz. Different amplifiers with separate grounds were used for each individual, optically coupled to the same computer. Given the possibility of mastoid asymmetries, data were re-referenced offline to an average of the left and right mastoid separately for each participant. EEG recordings were band-pass filtered from 0.5 to 70 Hz and corrected for eye movements using the Gratton and Coles algorithm [30]. This algorithm corrects ocular artifacts by subtracting the voltages of EOG channels scaled by the correction or propagation factor. The correction factor is estimated by linear least-square regression between EOG and EEG, whereby the EOG serves as the independent variable. As stated by Hoffmann and Falkenstein [31], regression-based eye correction methods (e.g., Gratton and Coles algorithm) provide comparable results to an independent component analysis (ICA) approach.

Spontaneous EEG activity was resampled at 250 Hz and divided into10-s epochs. Eye blink and movement artifacts were rejected based on a gradient criterion with a maximum admissible voltage step of 50 mV, and a difference criterion with a maximum admissible absolute difference of 200 mV between two values in a segment. A visual inspection in a semiautomatic mode was also performed. Only artifact-free epochs were chosen for further analyses. To reduce the amount of data and to overcome the problem of volume conduction between neighboring electrodes, we selected 21 electrodes based on the 10−20 system (Fp1, Fpz, Fp2, F7, F3, Fz, F4, F8, T7, C3, Cz, C4, T8, P7, P3, Pz, P4, P8, O1, Oz, and O2). These electrodes are distributed across the entire cortex, so that the information of the remaining electrodes would be rather redundant.

### Phase Synchronization (Coupling) Measures

**Figure 1:**
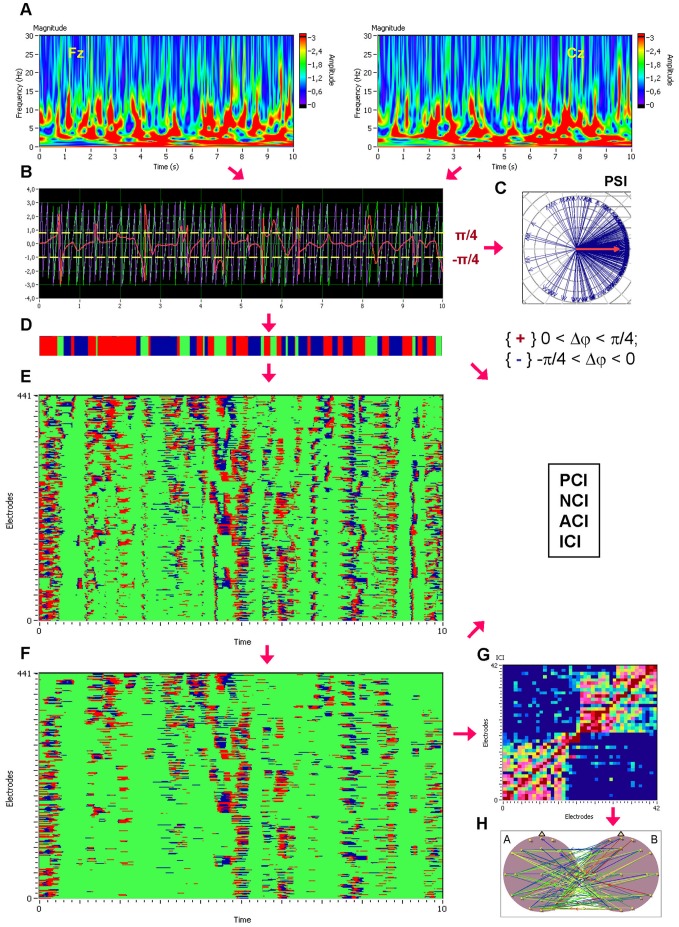
Schematic presentation of phase synchronization analyses. **A,** Complex Morlet wavelet transformation of signals from two channels (Fz and Cz) in time-frequency domain. **B,** Time course of instantaneous phases from these two channels and their phase difference (Fz = violet curve; Cz = green curve; Fz-Cz = red curve). **C,** The phase difference is depicted in form of the vectors in complex space, where the blue arrows reflect single phase angles and the red arrow represents the mean vector of the angular dispersions; its length displays the *PSI* measure. **D,** Coding of the phase difference of two signals, S1 (e.g., Fz) and S2 (e.g., Cz), at a given frequency (−π/4< S1–S2<0: blue stripes; 0< S1–S2< +π/4: red stripes; S1–S2< −π/4 or S1–S2> +π/4: green stripes = non-synchronization). **E,** Pair-wise synchronization pattern of the inter-brain network including all possible inter-brain connections from the one guitarist’s brain to the other (441 lines). Each line represents one pair of channels, such that there are 21 lines for each channel representing the coded phase difference between this channel and all other channels of the companion guitarist. Note that these phase differences coded with +1 (red), 0 (green), or –1 (blue) at each time point are used to calculate the four synchronization measures (i.e., *PCI, NCI, ACI,* and *ICI*) described in the Methods. **F,** The same synchronization pattern as in **E** after the cleaning procedure described in the text. **G,** Connectivity matrix of the joint network (42×42) with all significant intra- and inter-brain connections for the ICI (Integrative Coupling Index). **H,** Brain maps indicating significant connections between the brains.

To investigate phase coupling in a directed and frequency-resolved manner (cf. [7]), we applied an analytic or complex-valued Morlet wavelet transform to compute the instantaneous phase in the frequency range from 0 to 50 Hz in 0.002-Hz steps (see Figure 1A). The complex mother Morlet wavelet, also called Gabor wavelet, has a Gaussian shape around its central frequency *f*:
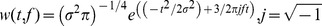
in which σ is the standard deviation of the Gaussian envelope of the mother wavelet. The wavelet coefficients were calculated with a time step of 5 leading to a time resolution of 20 ms and frequency resolution of 0.122 Hz. To identify the phase relations between any two channels, the instantaneous phase difference Δφ*_mn_*(*t, f*) was then computed from the wavelet coefficients for all possible electrode pairs within and between the brains (see Figure 1B).

Several different synchronization measures were obtained from these phase differences for frequency of interest *f_i_*. First, we obtained the Phase Synchronization Index (*PSI*), which is defined by
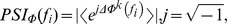
where 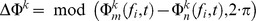 is the phase difference at the frequency *f_i_* between the instantaneous phases of the two signals across *k* data points in the segment; 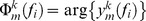 and 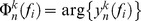. The *PSI* is similar to phase coherence, with the difference that the *PSI* measures phase stability or phase invariance *across time* within a trial or time series (e.g., [32]).

In addition to the *PSI* (see Figure 1C), which is independent from the phase angle in the sense that *PSI* can be high at different phase angles (phase angle differences), we calculated further synchronization indexes reflecting in-phase synchronization between two electrodes, that is, the extent to which the angle of phase differences approximates 0 [7]. Given the estimates of the phase difference between pairs of signals (channels), it is possible to ascertain how long the phase difference remains stable in defined phase angle boundaries by counting the number of points that are phase-locked at a defined time window. We adapted and slightly modified the procedure described in Kitzbichler et al. [33] in that we divided the range between −π/4 and +π/4 into two ranges and distinguished between positive and negative deviations from phase zero. As shown in Figure 1D, we marked negative deviations in the range between −π/4 and 0 in blue (coded with “−1”) and the positive deviations in the range between 0 and +π/4 in red (coded with “+1”). Phase difference values beyond these range were marked with green (coded with “0”) and represent non-synchronization. In the case of two channels, A and B, a blue stripe in the diagram would mean that the phase of channel B precedes the phase of channel A and a red stripe would mean that the phase of channel A precedes the phase of channel B. We then counted the number of data points that are phase-locked, separately, in each of these two ranges. Before counting, successive points in the defined range (between −π/4 and +π/4) with a time interval shorter than a period of the corresponding oscillation at the given frequency (*T_i_* = 1/*f_i_*) were discarded from the analysis. This cleaning procedure effectively eliminated instances of accidental synchronization (cf. Figures 1E and 1F). On the basis of this counting, we obtained several synchronization indices: (1) the Positive Coupling Index, *PCI*, or the relative number of phase-locked points in the positive range (between 0 and +π/4); (2) the Negative Coupling Index, *NCI*, or the relative number of phase-locked points in the negative range (between −π/4 and 0); (3) the Absolute Coupling Index, *ACI*, or the relative number of phase-locked points in the positive and negative range (i.e., between −π/4 and +π/4) indicating in-phase synchronization; (4) the Integrative Coupling Index, *ICI*, calculated by the formulae:
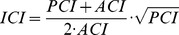



The *ICI* is equal to 1 when all points are phase-locked and positive; if all phase-locked points are negative, the term 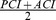 will approach 0.5, and, through multiplication with 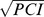, the *ICI* will approach 0. Thus, the *ICI* measure ranges from 0 and 1 and is asymmetric, (*ICI*_AB_ ≠ *ICI*_BA_) indicating the relative extent of positive phase synchronization [7]. These indexes were computed across all possible electrode pairs within and between the brains and are presented on a two-dimensional graph (Figure 1G) and brain maps (Figure 1H).

All previous coupling measures (*PCI*, *NCI*, and *ACI*) are related to all measurement points in the window and range from 0 and 1. *PSI* and *ACI* are symmetrical measures (i.e., *PSI*_AB_ = *PSI*_BA_ and *ACI*_AB_ = *ACI*_BA_) and have similar properties when synchronization is in phase. Positive and Negative Coupling Indexes are asymmetrical in the sense that *PCI*_AB_ ≠ *PCI*_BA_, however, (*PCI*_AB_+*NCI*_AB_) = –(*PCI*_BA_+*NCI*_BA_) or *PCI*_AB_ = |*NCI*_BA_|. The advantage of all these synchronization indexes, including the *PSI*, is that they measure synchronization in real time, and do not require comparisons across trials. Hence, they are suitable for analyzing continuous EEG signals.

To test and validate the described coupling measures (*PSI*, *ACI*, and *ICI*), we used the framework of “The Virtual Brain” developed by Viktor Jirsa and colleagues (TVB, www.thevirtualbrain.org; [34]–[35]). Simulation results showed that all three measures capture the intended coupling properties. These results are described in Text S1 and Figure S1. We restrict the description of our study results to the *ICI* measure, which is most informative due to its directionality. Results concerning *PSI* and *ACI* can also be found in the Text S1 and corresponding Supplementary Figures.

### Graph-Theoretical Approach (GTA)

#### Threshold determination

To determine the network properties, the thresholds of the synchronization or coupling measures were calculated first. For this purpose we generated surrogate data through a random permutation of available data points (“shuffling”) of all epochs at all channels included in the analyses, and then calculated the corresponding synchronization measures between all possible electrode pairs on the basis of these surrogate data. Thereafter, we applied a bootstrapping procedure with 1,000 resamples of the coupling measures obtained from the surrogate data set and determined the threshold as the bootstrapping mean plus the confidence interval at a significance level of p<0.0001 [36]–[38]. We used a high threshold level to obtain sparse networks with highly reliable connections. Only coupling values larger than the threshold value were considered as a link or edge in the given network.

#### Degrees and strengths

As *ICI* is a directed measure, we obtained the degree as the sum of in- and out-degrees, in which the in-degree is the sum of all incoming connections, 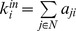, and the out-degree is the sum of all outgoing connections, 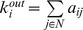. To calculate strengths, we then replaced the sum of the links with the sum of weights, 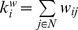. Thus, the strength can be considered as the weighted degree [20]. We determined degrees and strengths for the whole network of each guitarist pair and then calculated them for the within-brain network of each guitarist (A and B) separately. To exclusively determine the between-brain degrees and strengths, we subtracted degrees (and respectively strengths) of the within-brain network from the corresponding links of the network including both brains.

#### Clustering Coefficient (*CC*) and Characteristic Path Length (*CPL*)

If the nearest neighbors of a node are also directly connected to each other, they form a cluster. For an individual node, the *CC* is defined as the proportion of the existing number of connections to the total number of possible connections:
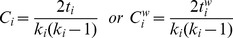
for binary and weighted networks, respectively, with 
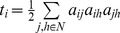
being the number of triangles around a node *i*. Usually, *C_i_* is averaged over all nodes to obtain a mean clustering coefficient (*CC*) of the graph:



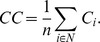



In the case of a directed graph, the mean *CC* is calculated by the formula:
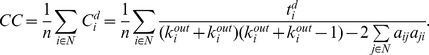



Random networks have low average clustering coefficients, whereas complex or small-world networks have high clustering coefficients (associated with high local efficiency of information transfer and robustness). Thus, the clustering coefficient is a measure of segregation.

Another important measure is the *CLP*. In the case of an unweighted graph, the shortest path length or distance *d_i,j_* between two nodes *i* and *j* is the minimal number of edges that have to be passed to go from *i* to *j*. This is also called the geodesic path between the nodes *i* and *j*. The *CLP* of a graph is the mean of the path lengths between all possible pairs of vertices:
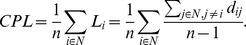



In the case of a weighted and directed graph the weight and direction of the links will be considered.

Random and complex real networks (or small-world networks, SWNs) have a short *CPL* (high global efficiency of parallel information transfer), whereas regular lattices have a long *CPL*. In this sense, *CPL* shows the degree of network integration, with a short *CPL* indicating higher network integration.

#### Small-worldness coefficients

To investigate the small-world properties of a network, it is common practice to compare its clustering coefficient and characteristic path length to those of regular lattices and random graphs. For this purpose, we constructed regular (lattice) and random networks that have the same number of nodes and the same mean degree as the observed networks. Random networks were constructed through randomization of the edges in the observed network. Lattice networks were configured like random networks, but edges were additionally redistributed after an initial random permutation such that they lie close to the main diagonal [39]. These network reconstructions for random and regular networks were carried out 20 times for each individual network. Average *CC* and *CPL* were then determined for these individual control networks.

Using these graph metrics, specific quantitative small-world metrics were obtained. The first small-world metric, the so-called small-world coefficient σ, is related to the main metrics of a random graph (*CC_rand_* and *CPL_rand_*) and is determined on the basis of two ratios 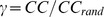 and 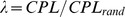:
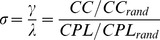



The small-world coefficient σ has been used in numerous networks showing small-world properties and has been found to be greater than 1 in the SWN (σ >1). The second small-world metric, the so-called small-world coefficient ω, is defined by comparing the clustering coefficient of the observed network to that of an equivalent lattice network and comparing the characteristic path length of the observed network to that of an equivalent random network:
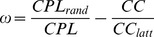



This metric is ranged between −1 and +1 and is close to zero for SWN (*CPL*_SWN_≈*CPL*_rand_ and *CC*_SWN_≈*CC*_latt_). Thereby, negative values indicate a graph with more regular properties (*CPL*_SWN_>>*CPL*_rand_ and *CC*_SWN_≈*CC*_latt_), and positive values of ω indicate a graph with more random properties (*CPL*_SWN_≈*CPL*_rand_ and *CC*_SWN_<<*CC_l_*_att_ ). The clear advantage of the metric ω compared to σ is the possibility to define how much the network of interest is like its regular or random equivalents [40].

#### Community structures and definition of node roles within the brain networks

To further investigate the topological properties of the hyper-brain networks, community structures for weighted undirected and directed networks, as well as indices of modularity (*M*), the within-module degree (*Z_i_*) and the participation coefficient (*P_i_*) were determined (cf. [20]). For this calculation, the modularity optimization methods [41], [42] were used, which are implemented in the Brain Connectivity Toolbox (https://sites.google.com/site/bctnet/; cf. [20]). The optimal community structure is a subdivision of the network into non-overlapping groups of nodes in a way that maximizes the number of within-module edges, and minimizes the number of between-module edges. The modularity (*M*) is a statistic that quantifies the degree to which the network may be subdivided into such clearly delineated groups or modules and is given for weighted networks by the formula [42]:
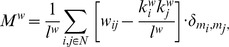
where 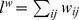 and 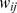 represents the weight of the edge between *i* and *j*, N is the total number of nodes in the network, 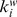 and 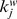 are weighted degrees or strengths of the nodes *i* and *j*, *m_i_* is the module containing node *i*, *m_j_* is the module containing node *j*, and 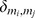 is the Kronecker delta, where 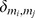 = 1 if *m_i_* = *m_j_*, and 0 otherwise. For directed unweighted networks, modularity was calculated in similar way by the formula [41]:



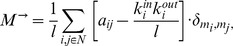
where 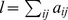 is the number of edges in the graph, and 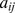 is defined to be 1 if there is an edge from *j* to *i* and zero otherwise, 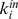 and 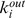 are the in- and out-degrees of the node *i*, and 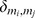 is again the Kronecker delta. High modularity values indicate strong separation of the nodes into modules. *M* = 0 if nodes are placed at random into modules or if all nodes are in the same cluster [43].

The within-module degree *Z_i_* indicates how well node *i* is connected to other nodes within the module m_i_. It is determined by
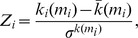
where *k_i_*(*m_i_*) is the within-module degree of node *i* (the number of links between *i* and all other nodes in *m_i_*). 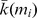 and 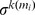 are the mean and standard deviation of the within-module degree distribution of *m_i_*. The within-module degree *Z_i_* is 0 if all the nodes of the module have the same number of edges (e.g., if all the nodes within the module are fully interconnected with each other); otherwise, it has negative or positive values depending on the number of links at the different nodes.

The participation coefficient *P_i_* describes how well the nodal connections are distributed across different modules:
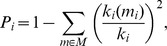



*M* is the set of modules, *k_i_(m_i_)* is the number of links between node *i* and all other nodes in module *m_i_*, and *k_i_* is the total degree of node *i* in the network. Correspondingly, *P_i_* of a node *i* is close to 1 if its links are uniformly distributed among all the modules and 0 if all of its links lie within its own module. *Z_i_*- and *P_i_*-values are characteristic for the different roles of the nodes in the network [43].

Following Guimerà and Amaral [43], we heuristically defined eight different universal roles, each characterized by *Z*- and *P*-values dividing the *Z-P* parameter space into eight different regions:

R1 (*Z*<1.4; *P*<0.05) – non-hub ultra-peripheral nodes,

R2 (*Z*<1.4; 0.05<*P*<0.5) – non-hub peripheral nodes,

R3 (*Z*<1.4; 0.5<*P*<0.8) – non-hub connector nodes,

R4 (*Z*<1.4; *P*>0.8) – non-hub kinless nodes,

R5 (*Z*>1.4; *P*<0.05) – hub ultra-peripheral nodes,

R6 (*Z*>1.4; 0.05<*P*<0.5) – hub peripheral nodes,

R7 (*Z*>1.4; 0.5<*P*<0.8) – hub connector nodes,

R8 (*Z*>1.4; *P*>0.8) – hub kinless nodes.

The community structures and corresponding measures (*M*, *Z_i_*, and *P_i_*) were determined for the joint networks of the two guitarists and for each individual brain separately. As the separate within-brain networks showed relatively low modularity values (*M* <0.30; cf. [44], [45]), we only report results for the joint networks. All GTA measures described here were determined using the Brain Connectivity Toolbox (cf. [20]) for 17 frequency components: 2, 3, 4, 5, 6, 7, 8, 9, 10, 11, 12, 14, 16, 18, 20, 24, and 28 Hz.

### EEG Data Reduction and Statistical Analyses

For statistical analyses, we collapsed frequencies into five bands: delta (2–3 Hz), theta (4–7 Hz), alpha (8–12 Hz), beta1 (14–20 Hz) and beta2 (24–28 Hz). Also, individual electrodes were collapsed into three regions along the anterior-posterior axis (frontal, central and parieto-occipital). Degrees and strengths were statistically evaluated for hyper-brain networks and separately for within- and between-brains connections. A four-way repeated-measures ANOVA with a between-subject factor Guitarist (guitarist A vs. guitarist B) and three within-subject factors Play Condition (Play A, Play B, and Play AB), Frequency Band (delta, theta, alpha, beta1, and beta2), and Site (frontal, central, and parietal) was used for all the coupling measures (*PSI*, *ACI*, and *ICI*). *CC, CPL*, and *M*, which were determined for hyper-brain networks only, were tested using a two-way repeated-measures ANOVA with two within-subject factors Play Condition and Frequency Band.

## Results

By simultaneously recording the EEG of two people, we measured the electrophysiological brain activity of eight pairs of guitarists during musical improvisation. The two guitarists were either playing simultaneously, or one of them was playing while the other was listening. A video recording of a pair of guitarists playing together with the corresponding EEG is available as a Movie S1. As mentioned in the Methods section, we restrict the description of our results to the directed *ICI* measure. Results regarding *PSI* and *ACI* can be found in the Supporting Information files. With a few exceptions, the pattern of results obtained with *PSI* and *ACI* matched the pattern of results obtained with *ICI*.

### Phase Synchronization and Brain Connectivity Patterns

**Figure 2:**
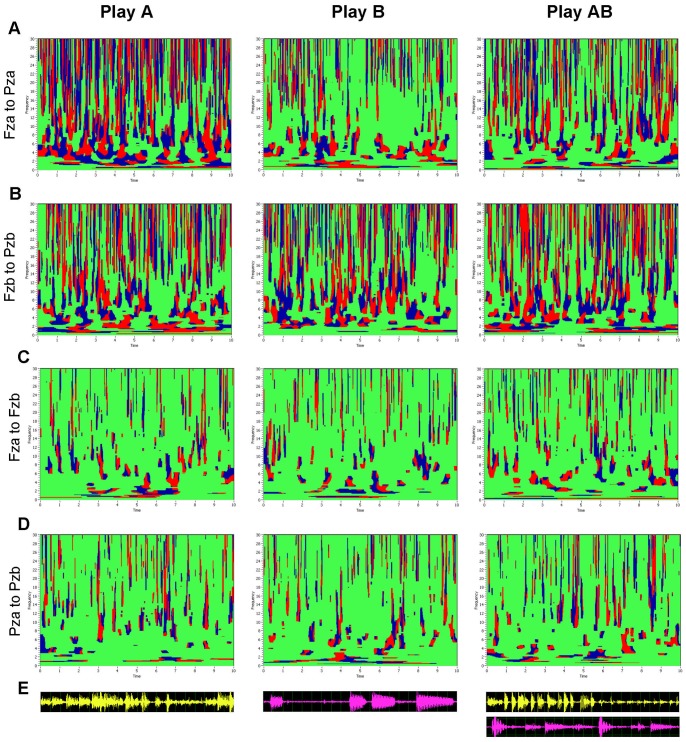
Phase synchronization patterns across frequencies (0–30 Hz) of one pair of guitarists under the three playing conditions (Play A, Play B, and Play AB). **A,** Phase synchronization patterns within the brain of guitarist A (Fza to Pza). **B,** Phase synchronization patterns within the brain of guitarist B (Fzb to Pzb). **C,** Phase synchronization patterns between the brains at the frontal electrode sites (Fza to Fzb). **D,** Phase synchronization patterns between the brains at the parietal electrode sites (Pza to Pzb). **E,** Microphone traces of guitar A (yellow) B (purple), respectively. Play A: Guitarist A is playing and guitarist B is listening; Play B: Guitarist B is playing and guitarist A is listening; Play AB: Both guitarist A and B are playing.

In Figure 2, we illustrate time-frequency diagrams of synchronization patterns between two electrodes within (Fza to Pza or Fzb to Pzb; see Figure 2A and 2B, respectively) and between (Fza to Fzb and Pza to Pzb, see Figure 2C and 2D, respectively) the brains in the frequency range between 0 and 30 Hz while (i) guitarist A is playing and guitarist B is listening; (ii) guitarist B is playing and guitarist A is listening; and (iii) both guitarists are playing together. It can be seen that in all these cases there is a complex interplay of synchronization patterns across frequencies and time, with the direction of temporal phase differences marked in blue and red, respectively. It is not surprising that synchronization within the brains is always stronger than that between the brains, but synchronization both within and between the brains seems to be strongly related to the plucked notes or chords that are played (compare Figure 2E). Furthermore, there is strong phase alignment across frequencies, which indicates coactivity between different networks operating in different frequency domains.

**Figure 3:**
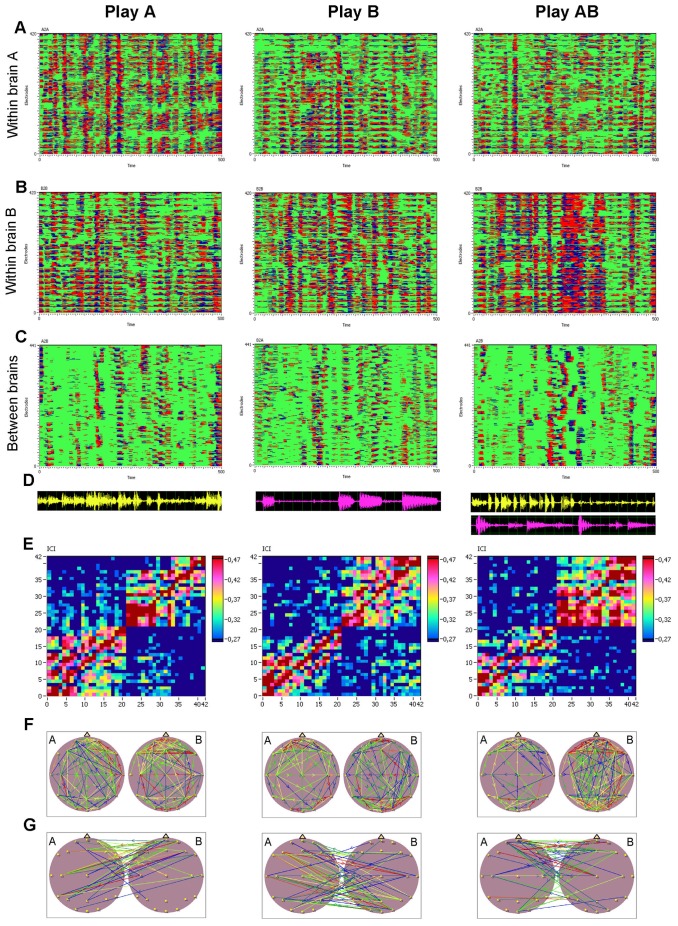
Phase synchronization patterns at the frequency of interest (6 Hz) for all possible electrode pairs within and between the brains under the three play conditions (Play A, Play B, and Play AB). **A,** Phase synchronization patterns within the brain of guitarist A. **B,** Phase synchronization patterns within the brain of guitarist B. **C,** Phase synchronization patterns between the brains of the guitarists A and B. **D,** Microphone traces of guitar A (yellow) and B (purple), respectively. **E,** Connectivity matrices of the joint network (42×42) with all significant intra- and inter-brain connections for the ICI (Integrative Coupling Index). **F,** Brain maps indicating significant connections within the brains. **G,** Brain maps indicating significant connections between the brains.

Within-brains synchronization patterns at the frequency of interest (*f_i_* = 6 Hz) across all possible electrode pairs for guitarists A and B are presented in Figures 3A and 3B, respectively. The between-brains synchronization pattern is depicted in Figure 3C. Corresponding recordings of acoustic channels for guitarists A and B as well as the AB duet are presented below in the respective synchronization patterns in Figure 3D. The connectivity matrices corresponding to the individual and duet synchronization patterns can be found in Figure 3E. The brain maps for *ICI* within and between the brains are presented in Figures 3F (within) and 3G (between). All panels in Figure 3 represent three playing conditions (Play A, Play B, and Play AB). For better visualization, brain maps containing the strongest within- and between-brain connections are depicted. Figure S2 shows the entire network with all significant connections within and between the brains.

Intra-brain connections were distributed across the entire cortex involving different brain regions or networks (prefrontal, motor, auditory, visual cortices, etc.) in both the playing and the listening guitarist, with slightly stronger interconnectivity within the playing guitarist’s brain. The inter-brain connectivity differed between the two guitarists: While guitarist A showed strong outgoing connections from frontal and central regions, the outgoing connections of guitarist B are distributed across different regions, with a relatively high number of connections going to frontal and occipital (e.g., Oz) electrodes of guitarist A. However, it should be noted here that connectivity patterns varied from pair to pair and across segments within pairs. To describe these changes in greater detail, we used the GTA.

### Statistical Evaluation of GTA Measures

#### Degrees and strengths

Strengths averaged across participants separately for the three play conditions and 21 electrode sites are represented in Figure S3 for some frequencies of interest (6, 10, 16 and 28 Hz). At higher frequencies (8 Hz and higher), strengths (in- and out-strengths) of the playing guitarists within their brains were higher than that of listening guitarists. This is particularly the case when playing solo. In addition, there were low strengths between the brains in the duet-playing condition in the alpha frequency range (e.g., 10 Hz), and high strengths between the brains in the beta1 frequency range, especially at 16 Hz.

For statistical analyses, we collapsed frequencies into five bands: delta (2–3 Hz), theta (4–7 Hz), alpha (8–12 Hz), beta1 (14–20 Hz) and beta2 (24–28 Hz). Also, individual electrodes were collapsed into three regions along the anterior-posterior axis (frontal, central and parieto-occipital). We obtained relatively similar results for degrees and strengths as well as in- and out-degrees/−strengths. Therefore, we only present out-strengths here, which are more informative than degrees since they account for the weights of the network links. The out-strengths were determined for the whole network of the duet, encompassing both within- and between-brain connections as well as separately for within- and between-brain networks. For the joint networks, a four-way repeated-measures ANOVA (Guitarist × Play Condition × Frequency Band × Site) for *ICI* revealed significant main effects of Frequency Band, F(4,56) = 28.2, P<0.0001, η^2^ = 0.67, and Site, F(2,28) = 28.0, P<0.0001, η^2^ = 0.67, indicating an increase in strengths with higher frequency and a decrease in strengths from frontal to parieto-occipital regions. Furthermore, significant interactions of Guitarist × Play Condition, F(2,28) = 4.0, P<0.05, η^2^ = 0.22, and Guitarist × Play Condition × Frequency Band, F(8,112) = 10.1, P<0.0001, η^2^ = 0.42, were found, indicating higher strengths in the playing guitarist compared to his or her listening partner. This difference was especially pronounced for both beta frequency bands (i.e., beta1 and beta2). When both guitarists were playing, there were no significant differences between them. Similar results were found for within-brain networks: Frequency Band, F(4,56) = 67.8, P<0.0001, η^2^ = 0.83; Site, F(2,28) = 24.4, P<0.0001, η^2^ = 0.64; Guitarist × Play Condition, F(2,28) = 4.1, P<0.05, η^2^ = 0.23; Guitarist × Play Condition × Frequency Band, F(8,112) = 11.4, P<0.0001, η^2^ = 0.45. In the between-brain network, however, statistical analyses of strengths showed a significant decrease of strengths with increasing frequency, indicated by the significant main effect Frequency Band, F(4,56) = 216.0, P<0.0001, η^2^ = 0.94, and the significant Frequency Band × Play Condition interaction, F(8,112) = 3.0, P<0.05, η^2^ = 0.18. This inverse relationship reflected variations in both within- and between-brains connections: *increasing* strength with higher frequency within the brains, and *decreasing* strength with higher frequency between the brains (see Table 1).

**Table 1:** Mean and standard deviation for strength of ICI measures calculated separately for intra- and inter-brain connections in the five frequency bands.

Frequency bands	Intra-brain	Inter-brain
Delta	7.42 (1.21)	2.28 (0.11)
Theta	8.05 (1.33)	1.86 (0.16)
Alpha	8.22 (1.50)	1.40 (0.13)
Beta 1	9.25 (1.39)	1.15 (0.09)
Beta 2	10.24 (1.43)	1.18 (0.14)

#### Clustering Coefficient (*CC*) and Characteristic Path Length (*CPL*)

*CC* and *CPL* were determined across all pairs of guitarists for different frequency bins and play conditions, taking into account intra- and inter-brain connections. Particular frequencies were then collapsed into the five frequency bands and analyzed using two-way repeated measures ANOVA (Play Condition × Frequency Band).

The rmANOVA showed a significant main effect of Frequency Band only, indicating significant changes across the frequency bands: *CC* increased with higher frequencies, F(4,28) = 158.8, P<0.0001, η^2^ = 0.96), and *CPL* decreased, at least for beta1 und beta2 frequency bands, F(4,28) = 29.6, P<0.0001, η^2^ = 0.81.

#### Small-worldness of hyper-brain networks

**Figure 4:**
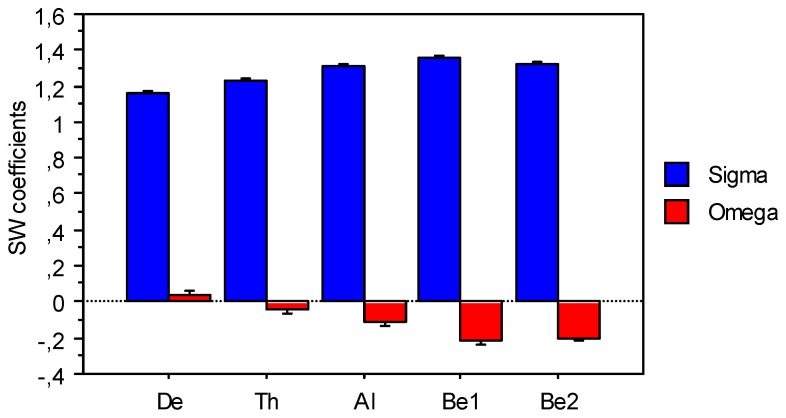
Small-worldness coefficients sigma (σ) and omega (ω) for the *ICI* measure in the five frequency bands (delta, theta, alpha, beta 1,and beta 2). The X-axis corresponds to the five frequency bands: De = Delta, Th = Theta, Al = Alpha, Be1 = Beta 1, and Be2 = Beta 2.

The small-world characteristics (σ and ω) for the *ICI* measure in the different frequency bands averaged across the three play conditions and all guitarist pairs are presented in Figure 4. According to the small-world coefficient σ, the *ICI*-based hyper-brain networks correspond to the SWN, wherein σ is always greater than 1 and increases with higher frequency. The small-world coefficient ω decreases with higher frequency and ranges between –0.22 and 0.05. This indicates that networks based on *ICI* possess more random characteristics for the delta frequency band and more regular characteristics for the beta frequency band. Statistical analysis revealed only a significant main effect of Frequency Band for both coefficients (σ and ω): σ, F(4,28) = 46.38, P<0.0001, η^2^ = 0.87), and ω, F(4,28) = 72.08, P<0.0001, η^2^ = 0.91).

#### Modularity, community structures and the *Z-P* parameter space

Modularity (*M*), which was analyzed in the same way as *CC* and *CPL,* showed a significant increase with frequency, F(4,28) = 178.1, P<0.0001, η^2^ = 0.96, indicating the stronger partitioning of networks synchronizing or communicating at high frequencies (e.g., beta frequency bands).

To define how nodes were positioned in their own module and with respect to other modules, we calculated the within-module degree (*Z_i_*) and participation coefficient (*P_i_*) of the node *i* for the whole network of a given pair. The within-module degree measures how ‘well-connected’ node *i* is to other nodes in the module, whereas the participation coefficient reflects how ‘well-distributed’ the links of the node *i* are among the other modules. *Z_i_* and *P_i_* form together the so-called *Z-P* parameter space, with different regions indicating specific roles of the nodes in this space or these regions.

**Figure 5:**
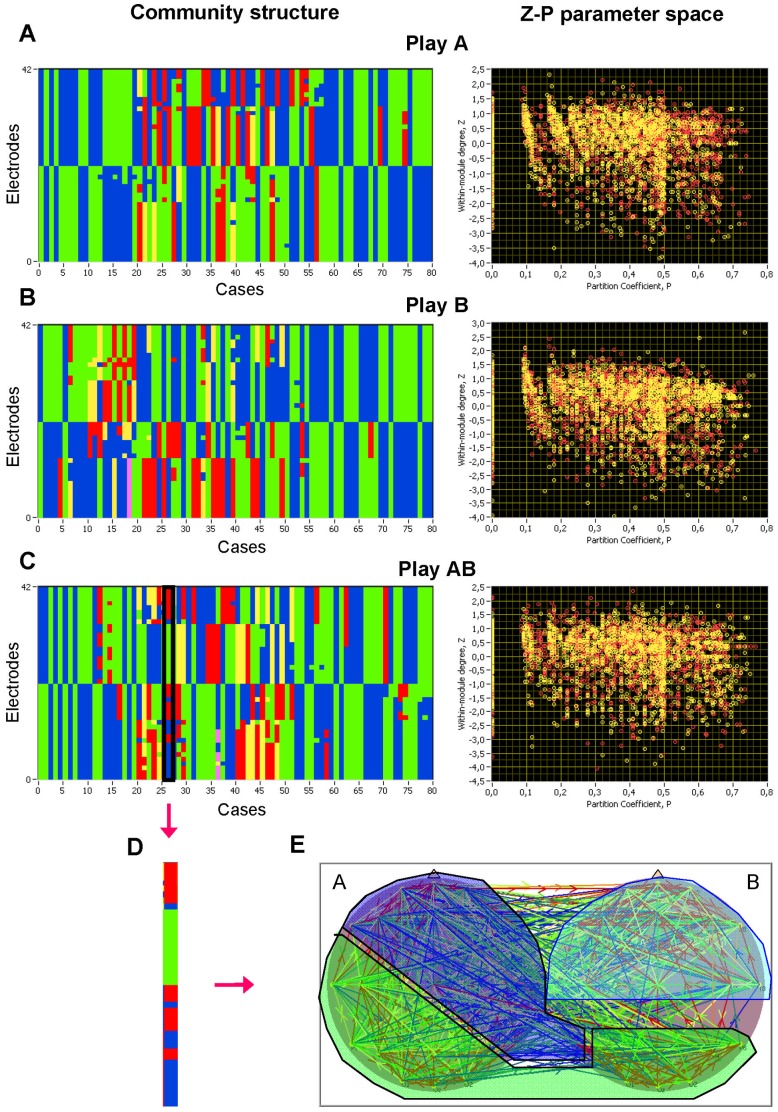
Community structures and distribution of nodes in a Z-P parameter space under the three play conditions (Play A, Play B, and Play AB). **A–C,** Community structure and *Z-P* parameter space for Play A, Play B and Play AB conditions. Community structure: In the X-axis (Cases), 10 trials of each of the 8 guitarist pairs are displayed. In the Y-axis (Electrodes), 21 electrodes of each guitarist in the pair are displayed. The color indicates the electrodes’ module affiliation. Z-P parameter space: The X- and Y-axes correspond to Partition coefficient (*P*) and Within-module degree (*Z*), respectively. Yellow circles display guitarist A, and red circles – guitarist B. **D–E,** A case in which some electrode sites from different brains share the same module, is displayed.

**Figure 6:**
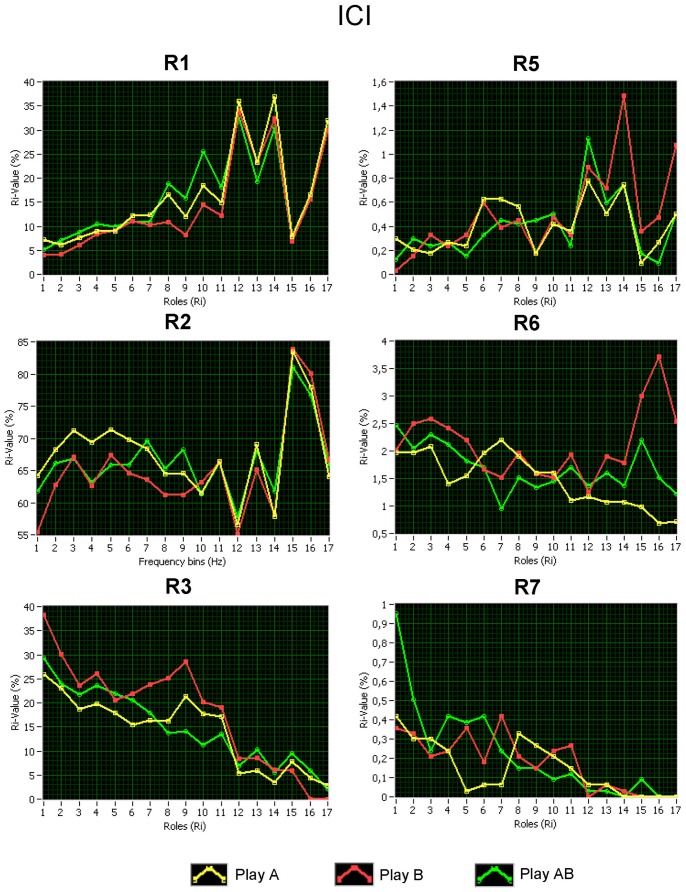
Changes in the number of nodes assuming different roles in the *Z-P* parameter space across the different frequencies for the ICI measure. Changes are presented for ultra-peripheral non-hubs (R1), peripheral non-hubs (R2), connector non-hubs (R3), and corresponding hubs (R5–R7) under the three conditions (Play A = yellow, Play B = red, and Play AB = green). Kinless hubs and non-hubs are excluded from the presentation because the number of nodes assuming these roles was very low. X-axis: Frequency bins; Y-axis: Percentage of number of nodes.

Figure 5 displays community structures and Z-P parameter spaces for the three play conditions (Play A, Play B, and Play AB) for all pairs of guitarists across the 10 different trials (see Figures 5A, 5B, and 5C). When comparing community structures at different frequencies (see Figure S4), we noticed that (i) the brains of the two guitarists being in different musical roles can always be differentiated independently of the frequency and the number of modules, (ii) the number of modules decreased with increasing frequency from about 3–4 (sometimes 5) in the delta and theta band to about 2–3 modules in the beta2 band, and (iii) there were cases at all frequencies in which some electrodes from different brains shared the same module (see Figures 5D and 5E). Regarding the *Z-P* parameter space, we noticed for all the measures a clear separation at *P* = 0.5 dividing the *Z-P* space into two regions: peripheral nodes (*P*≤0.5) and connectors (*P*>0.5). According to the within-module degree, nodes with *Z* ≥1.4 were defined as hubs and nodes with *Z* <1.4 were defined as non-hubs. This separation *Z*-value was chosen arbitrarily but it corresponds to the definition of hubs as nodes containing much more edges than the most of the nodes in the module (cf. [43]). The number of hubs varied between 0.6% and 2.8% for all the three measures at different frequencies. Depending on the participation coefficient, we further divided hubs and non-hubs into eight different roles: (R1) ultra-peripheral non-hubs (P≤0.05), (R2) peripheral non-hubs (0.05<P≤0.5), (R3) connector non-hubs (0.5<P≤0.8), (R4) kinless non-hubs (0.8<P≤1.0), and R5–R8 are then ultra-peripheral, peripheral, connector, and kinless hubs, respectively. We calculated the number of nodes falling into these regions. Results of this calculation for the *ICI* at different frequencies are displayed in Figure 6. It can be seen that the number of peripheral nodes, especially of ultra-peripheral nodes, increased with higher frequency, whereas the number of connectors decreased. These tendencies apply to both hubs and non-hubs. Interestingly, the low number of peripheral nodes was compensated by the high number of ultra-peripheral nodes, and vice versa (e.g., at the beta frequency 14–28 Hz). Furthermore, there was a higher number of non-hub connectors during separate playing (Play A and Play B) than during joint playing (Play AB) at the alpha frequency (8–12 Hz), whereas the joint playing was accompanied by a higher number of hub-connectors at the delta (2–3 Hz) and theta (5–7 Hz) frequency. We note that the present observations of differences between joint and separate playing are not backed up by inference statistics but are descriptive, and hence should be treated with caution. Please note that results regarding *ACI* and *PSI* measures are presented in Text S1, Tables S1 and S2, and Figures S5, S6, S7, and S8.

## Discussion

The primary objective of this study was to investigate the intra- and inter-brain dynamics in pairs of guitarists improvising together. In contrast to our previous studies [4], [6], where phase synchronization was measured across trials, we were interested in obtaining phase synchronization indices that can be applied to continuous data, or single trial analyses. Hence, we established directed and undirected synchronization or coupling measures derived from instantaneous changes in phase synchronization or phase differences between two EEG signals across time at different frequencies. These coupling indices measure different aspects of phase synchronization and are suitable for single-trial analysis (see also Text S1).

We observed phase synchronization patterns in the time-frequency domain and showed that intra- and inter-brain phase synchronization were operating at overlapping frequencies with different modes, such that the phase of a given signal at a given frequency can precede or follow another signal in phase. Generally, however, phase alignment across frequencies was a prominent feature of the data. Frequencies aligned in phase support the hypothesis that cell assemblies both within and between the brains synchronize during activities that require interpersonal action coordination in the time domain, such as musical improvisation. Phase-specific differences in phase state may suggest that the putative causal influence from one signal to the other is frequency dependent and may go in different directions at the same time.

### Graph-theoretical Measures: Degrees and Strengths

Next, we used graph-theoretical measures to describe the properties of intra- and inter-brain networks while improvising on the guitar in pairs. We found that (i) the strengths of outgoing connections increased with higher frequencies and were strongest at frontal sites, irrespective of the frequency, and (ii) the guitarist playing alone showed higher out-strengths than the listening guitarist, especially at the beta1 and beta2 frequencies, while no significant differences (between the two guitarists) were found when both guitarists were playing. Another interesting result was that strengths decreased with frequency in the between-brain networks, but increased with frequency in the within-brain networks. This inverse association suggests that intra-brain and inter-brain synchronization are operating at different modal frequencies.

In the study of Dumas et al. [3], three different inter-brain synchronization clusters among alpha-mu, beta and gamma frequency bands were found during spontaneous imitation episodes. In accordance with our findings, inter-brain synchronization measured by Phase Locking Value (PLV, a measure like *PSI* in our study) was higher in the alpha-mu frequency band than in the beta and gamma frequency bands. Furthermore, inter-brain synchronization comparing induced imitation episodes with “No View Motion” baseline could be found in the theta band only. The present results also replicate our earlier work, where inter-brain synchronization in guitarist duets as measured by Inter-Brain Phase Coherence (IPC) was predominantly observed in the delta and theta frequency bands [4], [6]. Taken together, the findings suggest a preponderance of low frequencies in inter-brain synchronization. Using the directed *ICI* measure, which reflects both strength and earliness in the phase angle, we were able to further specify this observation in the present study.

As indicated by a Frequency Band × Play Condition interaction for the inter-brain strengths, solo playing was accompanied by higher strengths in the alpha frequency range, while joint playing requires higher strengths in the beta1 frequency range (e.g., 16 Hz as shown in Figure S3). Joint playing is more of an interaction than solo playing and requires fast action coordination, which may be supported by faster frequencies. We have shown that inter-brain coupling generally prefers lower frequencies but faster frequencies may sometimes be required to support highly coordinative actions.

### Small-worldness, Segregation and Integration of Hyper-brain Networks

As a graph theory description of brain networks, we also computed the *CC* and the *CPL* as measures of segregation and integration, respectively [20], [46], [47]. We found that both the *CC* and *CPL* of the joint network of the two guitarists’ brains were frequency dependent. Whereas the *CC* increased with higher frequency, *CPL* decreased. Thus, directed networks measured by *ICI* showed stronger segregation and stronger integration at higher frequencies (e.g., beta1 and beta2). This tendency generalized across the two playing conditions.

To investigate the small-world properties of the hyper-brain networks, we compared their *CC* and *CPL* to those of regular lattices and random graphs. In general, random networks have a low average clustering coefficient, whereas complex or small-world networks have a high clustering coefficient (associated with the high local efficiency of information transfer and robustness). Random and small-world networks have a short *CPL* (high global efficiency of parallel information transfer), whereas regular networks (e.g., lattices) have a long *CPL*. To investigate small-world properties of the guitarists hyper-brain networks, we constructed regular and random networks with the same number of nodes and mean degree as our real networks and calculated two different small-worldness coefficients (i.e., σ and ω). According to the small-world coefficient σ, the directed hyper-brain networks correspond to SWN, whereby σ increases with higher frequency. This increase of σ for *ICI* with higher frequency corresponds to the increase of *CC* and decrease of *CPL* mentioned above. The fact that the small-worldness coefficient ω decreases with higher frequency and goes from positive (e.g., delta frequency band) to negative (especially for beta frequency band) indicates that *ICI*-based hyper-brain networks become more regular at higher frequencies. This seems to be a general tendency of the hyper-brain networks, because their small-world coefficients did not vary as a function of the play condition. In an MEG study, Stam [48] showed that binary unweighted networks correspond to SWNs for the delta and theta frequency bands, but are regular for the alpha and beta frequency bands. In an EEG study [49], where SWN properties were investigated using the synchronization measure at different frequencies, a decrease of small-worldness with higher frequency was also shown. The small-world index was not calculated by the authors, but can be obtained from changes in relative *CC* (γ) and *CPL* (λ); it approaches 1 at high frequencies. Other studies also have reported changes in network topology with a shift to more regular or more random networks, dependent on frequency or frequency bands [50]–[53]. There is also neuro-physiological evidence that small-world characteristics (e.g., CC and CPL or γ and λ) can vary as a function of age [47], [54], [55] or neuropathology [49], [51], [56]–[61], with a shift to a more regular or more random network topology (for a review, see [62] as well as [63]). According to a recent report, reduction in small-worldness is associated with a decrease in local efficiency and with an abnormal rise in interhemispheric connectivity [64]. Nevertheless, despite the currently available data on variations in small-worldness, our understanding of these differences is incomplete (see also results regarding *PSI* and *ACI* measures in Text S1). Also, it needs to be kept in mind that the results mentioned above all refer to single-brain studies (cf. [65]). Thus, even less is known regarding inter-brains networks. Further systematic research is needed to provide better understanding of this very important issue.

### Modular Properties of the Guitarists’ Networks

Modularity (*M*) measured for joint networks revealed a significant increase with higher frequencies, indicating a stronger partitioning of networks at higher frequencies. This stronger partitioning was accompanied by a reduced number of modules, with mostly two modules corresponding to the two brains of guitarist A and B, respectively (e.g., at beta2 frequency). As observed in our previous study [6], some nodes (electrode sites) that comprised in the same module belonged to different brains. The sharing of electrode sites from different brains in a common module suggests that the brain areas under these electrodes may participate in a function that engages both brains. Future research needs to determine whether and in what way these brain areas implement neuronal mechanisms of social interaction.

Next, we examined the *Z-P* parameter space of the joint network by looking at the within-module degree (*Z*) and the participation coefficient (*P*), which subdivide the *Z-P* parameter space into the eight regions corresponding to eight different roles of the nodes in the network: ultra-peripheral, peripheral, connectors, and kinless hubs, and corresponding non-hubs. Non-hub connectors are characterized by a low number of degrees or strengths within the module, and by a relatively high number of these between the modules. In contrast, hub-connectors have a relatively a high number of degrees or strengths both within and between the modules [43], [66]. We calculated the number of nodes assuming these different roles. A higher number of non-hub connectors during solo playing (Play A and Play B) as compared to joint playing (Play AB) could be found at the alpha frequency (8–12 Hz), whereas the joint playing was accompanied by higher number of hub-connectors at the delta (2–3 Hz) and theta (5–7 Hz) frequency. Thus, coordinated guitar playing during jazz improvisation increases the number of nodes having high connectivity within and between the modules. In light of the finding that strengths and degrees *between* brains increased at low frequencies, we suggest that the increase of connector hubs at low frequencies (e.g., delta and theta) during joint playing may be related, at least in part, to between-brain connections. In sum, we have shown that joint networks of guitarists’ duets during music/jazz improvisation, derived from connectivity analyses using different coupling measures, have a non-random modular organization, where the nodes or electrode sites have different functional roles within and between the modules, possibly contributing to interpersonal action coordination (e.g., playing guitar in duets).

### Limitations and Issues for Future Research

The present study has limitations and leaves room for questions to be addressed in future research. First, the sample size of our study was small. Further studies with larger samples would provide additional and more reliable information about coupling mechanisms during interpersonal action coordination. Second, certain features of our data-analytic approach to the study of network properties, such as the thresholding of coupling parameters and the definition of topological roles in *Z-P* parameter space, are somewhat arbitrary. Third, the synchronization measures used in this study reflect 1∶1 synchronization or synchronization at a given frequency. Relative (i.e., *n:m*) as well as nonlinear (or weak) synchronization [67], [68] may also fulfill important functions during interpersonal action coordination and should be investigated in the future. Cross-frequency coupling between low and high frequencies may be of particular importance, given that these frequencies are differentially related to the coupling dynamics within and between brains, as shown in the present study. Finally, and perhaps most importantly, analyses of intra- and inter-brain synchronization, such as the ones reported in this article, need to be combined with detailed observations of the microstructure of task-relevant behavior (e.g., gestures, eye movements) to better understand how inter-brain synchronization is initiated and how temporally specified expectations about the other person’s actions are updated.

## Conclusions

Extending a previous experimental design [4], [6], we found that within- and between-brain oscillatory couplings can also be observed during musical improvisation on the guitar. Furthermore, these couplings were also present when one guitarist was playing and the other was listening.

We found that synchronization patterns during guitar improvisation show a complex interplay of different frequencies. With increasing frequencies, strengths increased in within-brain networks, but decreased in between-brain networks, suggesting that intra-brain networks tend to operate at higher frequencies, whereas inter-brain networks tend to operate at lower frequencies. Furthermore, we found that the guitarist playing solo showed higher out-strengths than the listening guitarist at higher frequencies, while no significant differences (between the two guitarists) were found when both guitarists were playing. Joint playing was also accompanied by higher strengths in the beta1 frequency range, whereas solo playing was accompanied by higher strengths in the alpha frequency range.

The inspection of community structures showed a higher number of non-hub connectors during solo playing at the alpha frequency (8–12 Hz), whereas the joint playing was accompanied by a higher number of hub-connectors at the delta (2–3 Hz) and theta (5–7 Hz) frequency. We also found that hyper-brain network topology is frequency-dependent and approximates regular networks with increasing frequency, independent of play condition. Finally, we identified modules composed of nodes from both brains, so-called hyper-brain modules. The areas captured by these nodes may point to brain regions that implement mechanisms of interpersonal action coordination.

## Supporting Information

Figure S1 **Results of** ***PSI*** **,** ***ACI*** **, and** ***ICI*** **distributions for simulated data at the three different frequencies. A–C,** In this simulation, 5, 10, and 20 Hz oscillations with additive noise were used. The oscillations were divided pairwise into epochs of 3,000 ms, thereby the second oscillation in the pair was randomly shifted in phase, with a uniform distribution between –π and +π. The coupling (*PSI*, *ACI*, and *ICI*) was determined for 10,000 such epochs in total. Note that all three phase synchronization measures capture the intended coupling properties (see text for details).
(TIF)

Figure S2 **Network patterns and corresponding intra- and inter-brain maps at the frequency of interest (6 Hz) for the three coupling measures (** ***PSI*** **,** ***ACI*** **and** ***ICI*** **) under the three play conditions. A,** Connectivity matrices of the joint network (42×42) with all significant intra- and inter-brain connections for the *PSI* (Phase Synchronization Index). **B,** Brain maps indicating significant connections (*PSI*) within the brains. **C,** Brain maps indicating significant connections (*PSI*) between the brains. **D,** Connectivity matrices of the joint network (42×42) with all significant intra- and inter-brain connections for the *ACI* (Absolute Coupling Index). **E,** Brain maps indicating significant connections (*ACI*) within the brains. **F,** Brain maps indicating significant connections (*ACI*) between the brains. **G,** Connectivity matrices of the joint network (42×42) with all significant intra- and inter-brain connections for the *ICI* (Absolute Coupling Index). **H,** Brain maps indicating significant connections (*ICI*) within the brains. **I,** Brain maps indicating significant connections (*ICI*) between the brains.
(TIF)

Figure S3 **Out- and In-Strengths within and between the brains separately for guitarist A and B at some frequencies of interest (6, 10, 16 and 28 Hz) for the ICI measure under the three play conditions (Play A, Play B, and Play AB).** The X-axis represents 21 electrode (Fp1, Fpz, Fp2, F7, …, O1, Oz, and O2) of each participant. The different colours represent the play conditions: Play A = red, Play B = green, and Play AB = blue.
(TIF)

Figure S4 **Community structures for the ICI measure at some frequencies of interest (2, 10, 16 and 28 Hz) under the three play conditions (Play A, Play B, and Play AB).** In the X-axis (Cases), 10 trials of each of the 8 guitarist pairs are displayed. In the Y-axis (Electrodes), 42 electrodes (21 of each guitarist in the pair) are displayed. The color indicates the electrodes’ module affiliation.
(TIF)

Figure S5 **Strengths within and between the brains separately for guitarist A and B at some frequencies of interest (6, 10, 16 and 28 Hz) for the two undirected measures** ***PSI*** **and** ***ACI*** **under the three play conditions (Play A, Play B, and Play AB).** The X-axis represents 21 electrode (Fp1, Fpz, Fp2, F7, …, O1, Oz, and O2) of each participant. The different colours represent the play conditions: Play A = red, Play B = green, and Play AB = blue.
(TIF)

Figure S6 **Small-worldness coefficients σ and ω for** ***ACI*** **and** ***PSI*** **measures in the five frequency bands (delta, theta, alpha, beta 1,and beta 2). A,** Small-worldness coefficients σ and ω for *ACI*. **B,** Small-worldness coefficients σ and ω for *PSI*. The X-axis corresponds to the five frequency bands: De = Delta, Th = Theta, Al = Alpha, Be1 = Beta 1,and Be2 = Beta 2.
(TIF)

Figure S7 **Changes in the number of nodes assuming different roles in the** ***Z-P*** **parameter space across the different frequencies for the** ***PSI*** **measure.** Changes are presented for ultra-peripheral non-hubs (R1), peripheral non-hubs (R2), connector non-hubs (R3) and corresponding hubs (R5–R7) under the three conditions (Play A = yellow, Play B = red, and Play AB = green). Kinless hubs and non-hubs are excluded from the presentation because the number of nodes assuming these roles was very low. X-axis: Frequency bins; Y-axis: Percentage of number of nodes.
(TIF)

Figure S8 **Changes in the number of nodes assuming different roles in the** ***Z-P*** **parameter space across the different frequencies for the** ***ACI*** **measure.** Changes are presented for ultra-peripheral non-hubs (R1), peripheral non-hubs (R2), connector non-hubs (R3) and corresponding hubs (R5–R7) under the three conditions (Play A = yellow, Play B = red, and Play AB = green). Kinless hubs and non-hubs are excluded from the presentation because the number of nodes assuming these roles was very low. X-axis: Frequency bins; Y-axis: Percentage of number of nodes.
(TIF)

Table S1 Mean and standard deviation for strength of *ACI* and *PSI* measures calculated separately for intra- and inter-brain connections in the five frequency bands.
(DOCX)

Table S2 ANOVA results (F, P, and η^2^ values) for strength of *ACI* and *PSI* measures calculated for hyperbrain, intrabrain and interbrain connections.
(DOCX)

Text S1 **This text describes validation of the coupling measures on the simulation and results of the** ***ACI*** **and** ***PSI*** **measures.**
(DOCX)

Movie S1 File name: Video S1.mp4. File format: mp4. Description of data: This movie shows the simultaneous video and EEG recordings of a pair of guitarists playing an improvisation. EEG from 15 channels (F7, F3, Fz, F4, F8, T7, C3, Cz, C4, T8, P7, P3, Pz, P4, and P8) of both guitarist (A and B) are shown.
(MP4)
