# Supplementary figures and images for: Intra- and Inter-Brain Synchronization during Musical Improvisation on the Guitar
Source: PLoS One. 2013 Sep 10;8(9):e73852. doi: 10.1371/journal.pone.0073852 (PMC3769391; doi:10.1371/journal.pone.0073852)

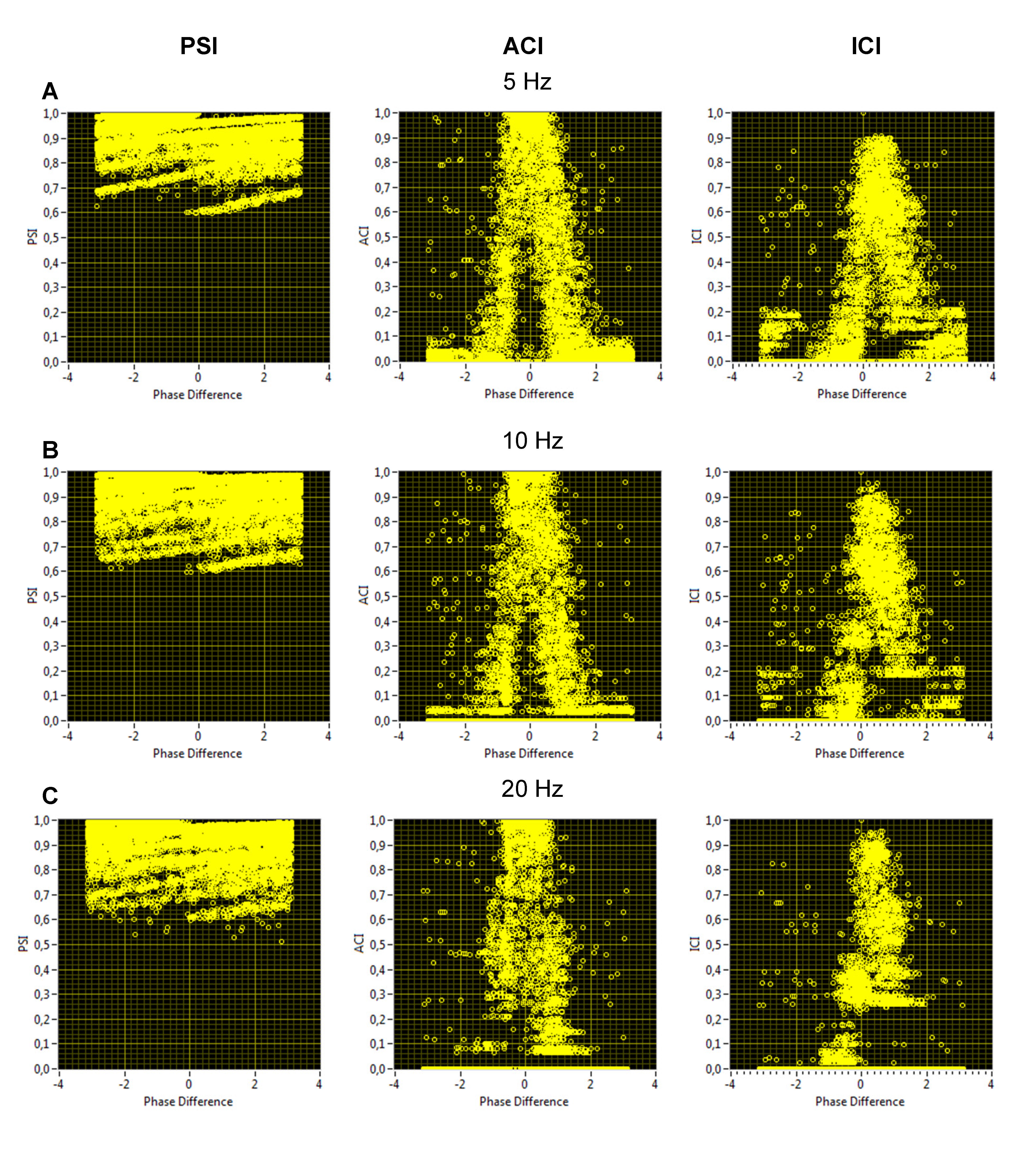

Supplement: Figure S1 — Results of PSI , ACI , and ICI distributions for simulated data at the three different frequencies. A–C, In this simulation, 5, 10, and 20 Hz oscillations with additive noise were used. The oscillations were divided pairwise into epochs of 3,000 ms, thereby the second oscillation in the pair was randomly shifted in phase, with a uniform distribution between –π and +π. The coupling (PSI, ACI, and ICI) was determined for 10,000 such epochs in total. Note that all three phase synchronization measures capture the intended coupling properties (see text for details). (TIF) [file pone.0073852.s001.tif]

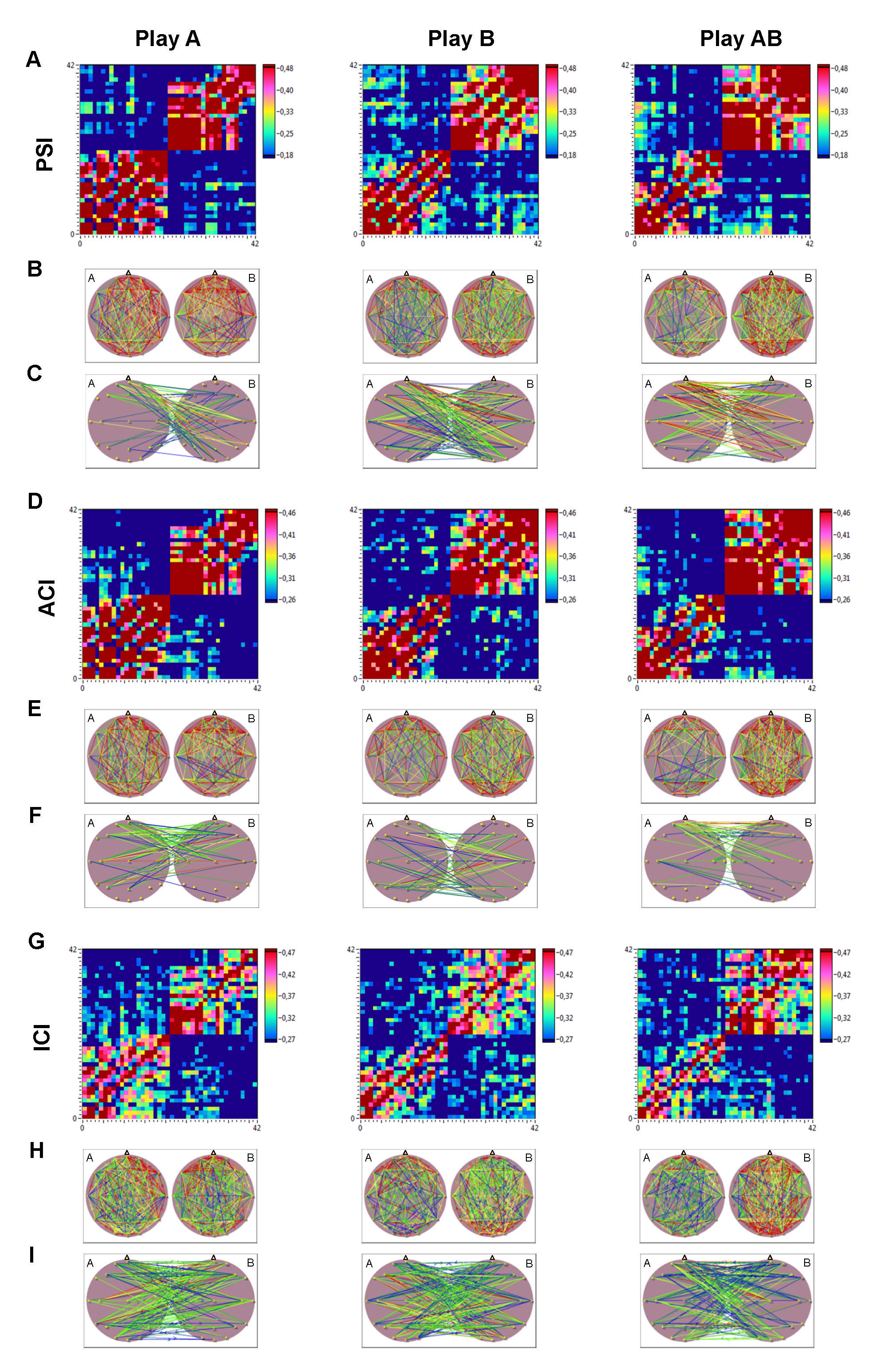

Supplement: Figure S2 — Network patterns and corresponding intra- and inter-brain maps at the frequency of interest (6 Hz) for the three coupling measures ( PSI , ACI and ICI ) under the three play conditions. A, Connectivity matrices of the joint network (42×42) with all significant intra- and inter-brain connections for the PSI (Phase Synchronization Index). B, Brain maps indicating significant connections (PSI) within the brains. C, Brain maps indicating significant connections (PSI) between the brains. D, Connectivity matrices of the joint network (42×42) with all significant intra- and inter-brain connections for the ACI (Absolute Coupling Index). E, Brain maps indicating significant connections (ACI) within the brains. F, Brain maps indicating significant connections (ACI) between the brains. G, Connectivity matrices of the joint network (42×42) with all significant intra- and inter-brain connections for the ICI (Absolute Coupling Index). H, Brain maps indicating significant connections (ICI) within the brains. I, Brain maps indicating significant connections (ICI) between the brains. (TIF) [file pone.0073852.s002.tif]

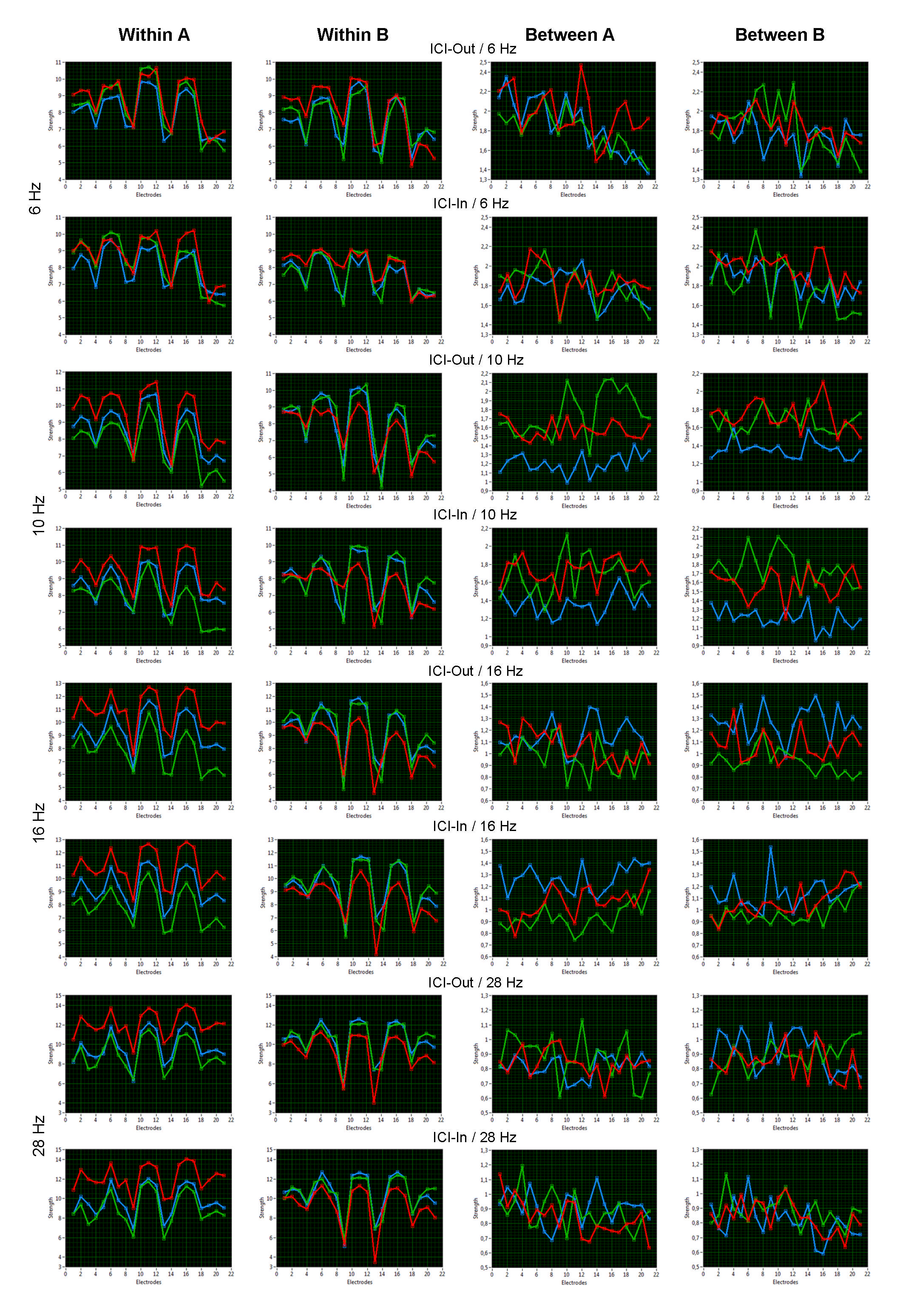

Supplement: Figure S3 — Out- and In-Strengths within and between the brains separately for guitarist A and B at some frequencies of interest (6, 10, 16 and 28 Hz) for the ICI measure under the three play conditions (Play A, Play B, and Play AB). The X-axis represents 21 electrode (Fp1, Fpz, Fp2, F7, …, O1, Oz, and O2) of each participant. The different colours represent the play conditions: Play A = red, Play B = green, and Play AB = blue. (TIF) [file pone.0073852.s003.tif]

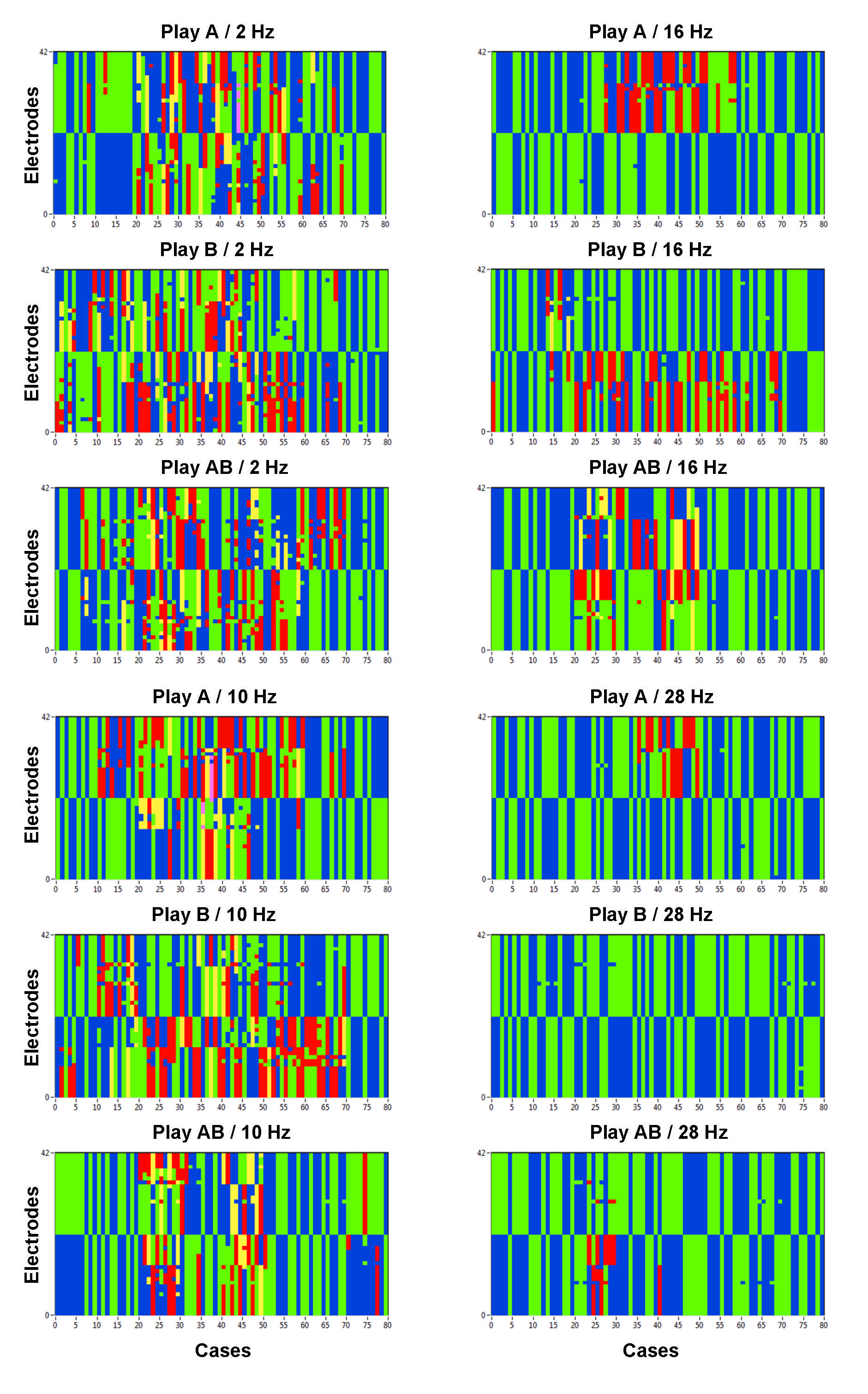

Supplement: Figure S4 — Community structures for the ICI measure at some frequencies of interest (2, 10, 16 and 28 Hz) under the three play conditions (Play A, Play B, and Play AB). In the X-axis (Cases), 10 trials of each of the 8 guitarist pairs are displayed. In the Y-axis (Electrodes), 42 electrodes (21 of each guitarist in the pair) are displayed. The color indicates the electrodes’ module affiliation. (TIF) [file pone.0073852.s004.tif]

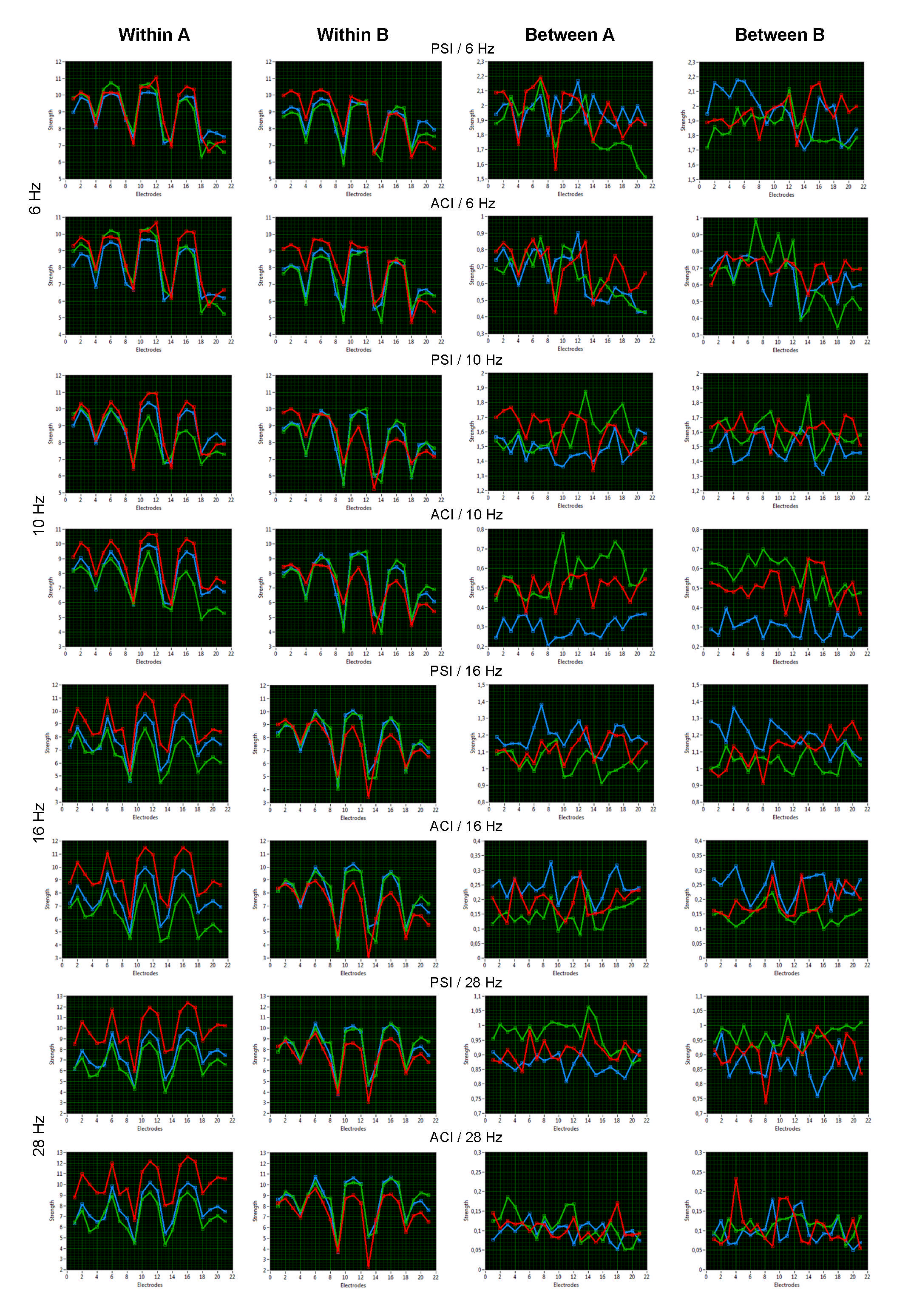

Supplement: Figure S5 — Strengths within and between the brains separately for guitarist A and B at some frequencies of interest (6, 10, 16 and 28 Hz) for the two undirected measures PSI and ACI under the three play conditions (Play A, Play B, and Play AB). The X-axis represents 21 electrode (Fp1, Fpz, Fp2, F7, …, O1, Oz, and O2) of each participant. The different colours represent the play conditions: Play A = red, Play B = green, and Play AB = blue. (TIF) [file pone.0073852.s005.tif]

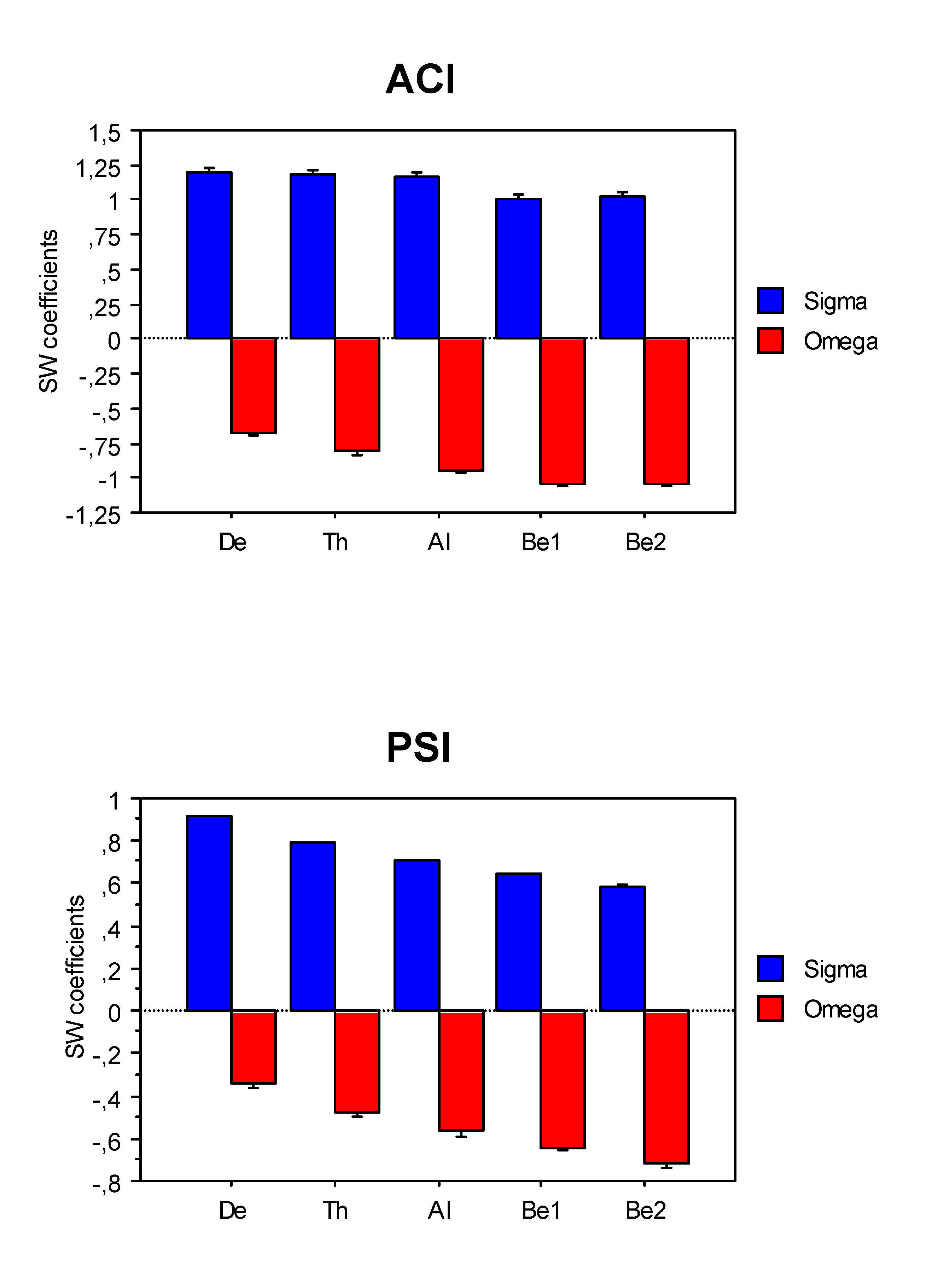

Supplement: Figure S6 — Small-worldness coefficients σ and ω for ACI and PSI measures in the five frequency bands (delta, theta, alpha, beta 1,and beta 2). A, Small-worldness coefficients σ and ω for ACI. B, Small-worldness coefficients σ and ω for PSI. The X-axis corresponds to the five frequency bands: De = Delta, Th = Theta, Al = Alpha, Be1 = Beta 1,and Be2 = Beta 2. (TIF) [file pone.0073852.s006.tif]

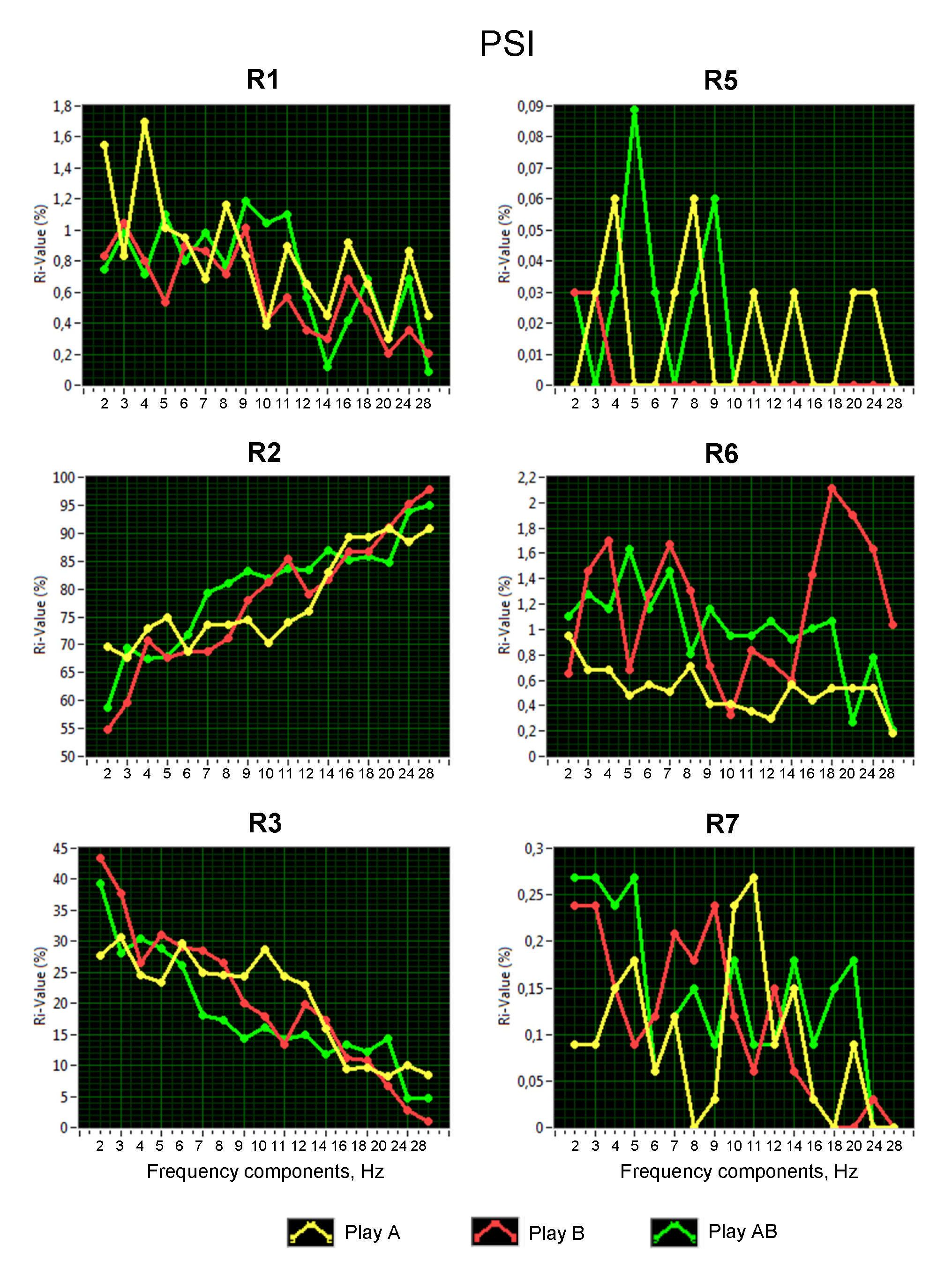

Supplement: Figure S7 — Changes in the number of nodes assuming different roles in the Z-P parameter space across the different frequencies for the PSI measure. Changes are presented for ultra-peripheral non-hubs (R1), peripheral non-hubs (R2), connector non-hubs (R3) and corresponding hubs (R5–R7) under the three conditions (Play A = yellow, Play B = red, and Play AB = green). Kinless hubs and non-hubs are excluded from the presentation because the number of nodes assuming these roles was very low. X-axis: Frequency bins; Y-axis: Percentage of number of nodes. (TIF) [file pone.0073852.s007.tif]

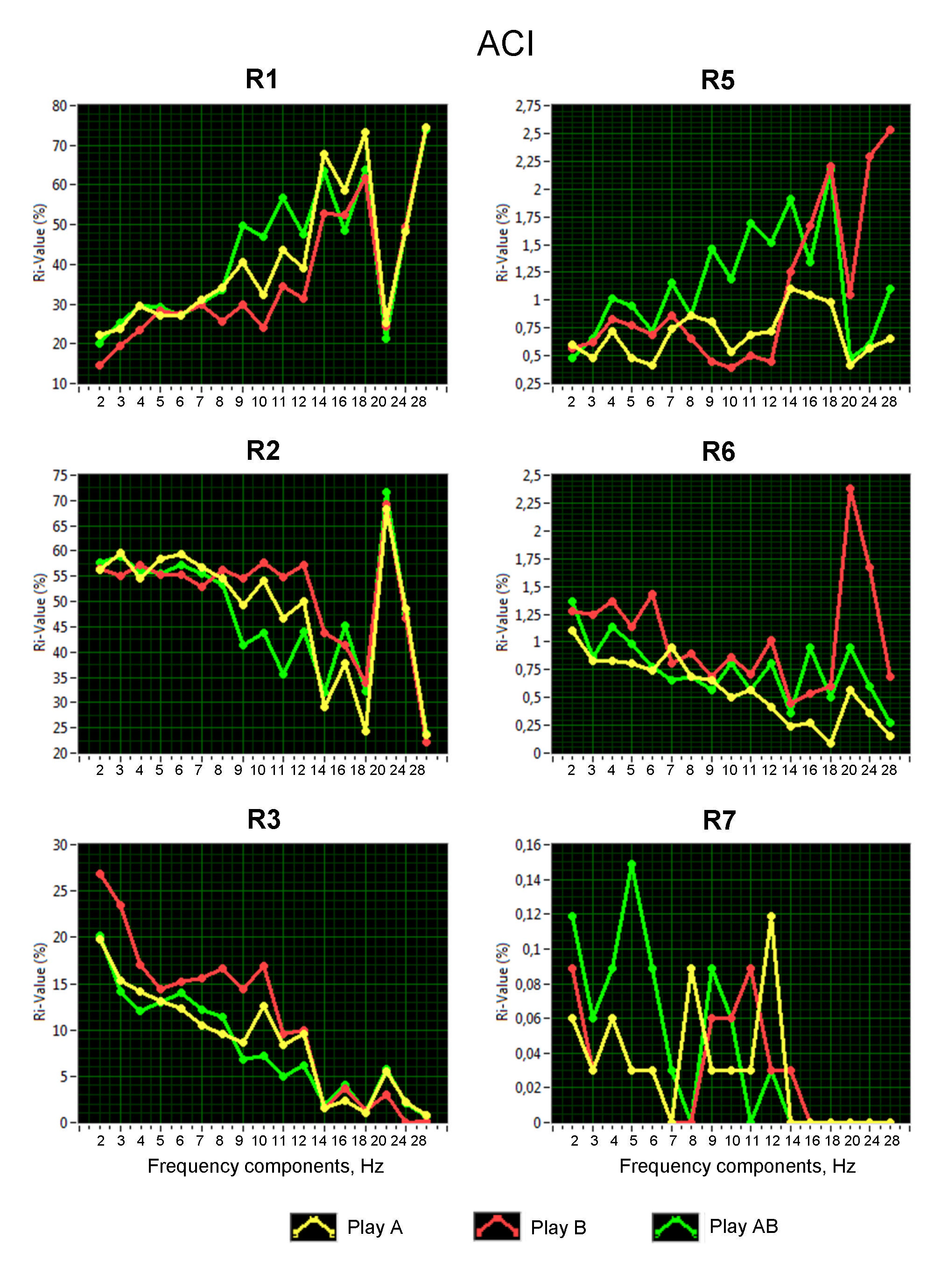

Supplement: Figure S8 — Changes in the number of nodes assuming different roles in the Z-P parameter space across the different frequencies for the ACI measure. Changes are presented for ultra-peripheral non-hubs (R1), peripheral non-hubs (R2), connector non-hubs (R3) and corresponding hubs (R5–R7) under the three conditions (Play A = yellow, Play B = red, and Play AB = green). Kinless hubs and non-hubs are excluded from the presentation because the number of nodes assuming these roles was very low. X-axis: Frequency bins; Y-axis: Percentage of number of nodes. (TIF) [file pone.0073852.s008.tif]
