# Supplementary material for: Intra- and Inter-Brain Synchronization during Musical Improvisation on the Guitar
Source: PLoS One. 2013 Sep 10;8(9):e73852. doi: 10.1371/journal.pone.0073852 (PMC3769391; doi:10.1371/journal.pone.0073852)
Supplement: Table S1 — Mean and standard deviation for strength of ACI and PSI measures calculated separately for intra- and inter-brain connections in the five frequency bands. (DOCX) [file pone.0073852.s009.docx]

**Table S1.** Mean and standard deviation for strength of *ACI* and *PSI* measures calculated separately for intra- and inter-brain connections in the five frequency bands.

| Frequency bands | *ACI* | | *PSI* | |
| --- | --- | --- | --- | --- |
|  | Intra-brain | Inter-brain | Intra-brain | Inter-brain |
| Delta | 7.71 (1.61) | 0.92 (0.11) | 9.15 (1.38) | 2.81 (0.18) |
| Theta | 7.87 (1.53) | 0.64 (0.10) | 8.82 (1.24) | 2.02 (0.10) |
| Alpha | 7.58 (1.47) | 0.39 (0.08) | 8.43 (1.16) | 1.51 (0.08) |
| Beta 1 | 7.69 (1.28) | 0.28 (0.04) | 7.83 (1.07) | 1.13 (0.05) |
| Beta 2 | 8.31 (1.38) | 0.23 (0.05) | 7.90 (1.27) | 0.93 (0.06) |

*ACI*=Absolute Coupling Index; *PSI*=Phase Synchronization Index.
