# Supplementary material for: Intra- and Inter-Brain Synchronization during Musical Improvisation on the Guitar
Source: PLoS One. 2013 Sep 10;8(9):e73852. doi: 10.1371/journal.pone.0073852 (PMC3769391; doi:10.1371/journal.pone.0073852)
Supplement: Table S2 — ANOVA results (F, P, and η2 values) for strength of ACI and PSI measures calculated for hyperbrain, intrabrain and interbrain connections. (DOCX) [file pone.0073852.s010.docx]

**Table S2.** ANOVA results (F, P, and η^^ values) for strength of *ACI* and *PSI* measures calculated for hyperbrain, intrabrain and interbrain connections.

| Effects (df) | *ACI* | | | *PSI* | | |
| --- | --- | --- | --- | --- | --- | --- |
|  | F | P | η^^ | F | P | η^^ |
| Hyperbrain connections | | | | | | |
| FB  (4,56) | 4.98 | 0.025 | 0.26 | 92.74 | 0.0001 | 0.87 |
| Site  (2,28) | 17.70 | 0.0001 | 0.56 | 14.75 | 0.0001 | 0.51 |
| FB x S (8,112) | 29.80 | 0.0001 | 0. 68 | 26.14 | 0.0001 | 0. 65 |
| FB x S x G (8,112) | 3.93 | 0.024 | 0.22 | 3.88 | 0.026 | 0.22 |
| FB x PC x G (8,112) | 9.68 | 0.0001 | 0.41 | 9.83 | 0.0001 | 0.41 |
| Intrabrain connections | | | | | | |
| FB  (4,56) | 4.06 | 0.045 | 0.23 | 18.07 | 0.0001 | 0.56 |
| Site  (2,28) | 15.85 | 0.0001 | 0.56 | 13.81 | 0.0001 | 0.50 |
| FB x S (8,112) | 29.39 | 0.0001 | 0.68 | 24.91 | 0.0001 | 0. 65 |
| FB x S x G (8,112) | 4.31 | 0.017 | 0.24 | 4.00 | 0.022 | 0.22 |
| FB x PC x G (8,112) | 9.53 | 0.0001 | 0.41 | 10.29 | 0.0001 | 0.42 |
| Interbrain connections | | | | | | |
| FB  (4,56) | 184.01 | 0.0001 | 0.93 | 855.02 | 0.0001 | 0.98 |
| Site  (2,28) | 4.26 | 0.046 | 0.23 | 1.81 | 0.19 | 0.12 |
| FB x S (8,112) | 2.81 | 0.019 | 0.17 | 3.39 | 0.021 | 0. 20 |
| FB x PC (8,112) | 3.41 | 0.034 | 0.20 | 3.28 | 0.028 | 0.19 |

FB = Frequency Band, S = Site, PC = Play Condition, and G = Guitarist.
