# Supplementary material for: Intra- and Inter-Brain Synchronization during Musical Improvisation on the Guitar
Source: PLoS One. 2013 Sep 10;8(9):e73852. doi: 10.1371/journal.pone.0073852 (PMC3769391; doi:10.1371/journal.pone.0073852)
Supplement: Text S1 — This text describes validation of the coupling measures on the simulation and results of the ACI and PSI measures. (DOCX) [file pone.0073852.s011.docx]

**Supplementary Text S1**

*Validation of the coupling measures on the simulation*

To test and validate the coupling measures *PSI*, *ACI*, and *ICI*, we used the framework of ‘The Virtual Brain’ (TVB, [www.thevirtualbrain.org](http://www.thevirtualbrain.org); see [34–35]). We simulated 5, 10, and 20 Hz oscillations provided with additive noise. We then divided these oscillations pairwise into epochs of 3000 ms, thereby shifting the second oscillation in the pair randomly in phase, with a uniform distribution between –π and + π. Altogether 10,000 epochs were produced in the simulation. Thereafter, all three coupling measures (*PSI*, *ACI*, and *ICI*) were determined for each epoch and corresponding oscillation pair. Results of this computation for the three frequencies of interest are presented in Supplementary Figure S1 (panels A, B, and C for 5, 10, and 20 Hz, respectively). It can be seen that *PSI* is equally distributed between between 0.60 and 1.00 for all three frequencies. In the case of *PSI*, the phase shift or time delay between the two time series does not affect coupling strength, as long as it remains constant, and should be equal to 1 for pure sinusoids. The observed smearing of *PSI* values is due to noise that disturbs the phases of the signal. *ACI* is an absolute in-phase coupling index and correspondingly shows a peak at 0 ± π/4, as it would be expected from the definition (see Methods for details). *ICI* is also an in-phase coupling index, which is oriented on positive phase shift in the interval between 0 and π/4 (i.e., *ICI* is maximal, if the phase difference between two signals is in the mentioned interval between 0 and π/4). Correspondingly, it shows a peak in the middle of this interval at π/8 ± π/4. In sum, the simulation results show that all three phase synchronization measures used in the study capture the intended coupling properties.

*Brain connectivity patterns for PSI and ACI measures*

Supplementary Figure S2 shows significant connections within and between the brains for all three measures (PSI, ACI, and ICI) in the three playing conditions (Play A, Play B, and Play AB) at the frequency of interest (f_i_ = 6 Hz). Panels A, D, and G show network matrices; panels B, E, and H show brain maps of connections within the brains, and panels C, F, and I show brain maps of connections between the brains. Intra-brain connections were distributed across the entire cortex involving different brain regions or networks (prefrontal, motor, auditory, visual cortices etc.) in both the playing and the listening guitarist, with slightly stronger interconnectivity within the playing guitarist’s brain. During joint playing, guitarist B in the pair displayed in Figure S2 showed stronger within-brain connectivity than guitarist A. Inter-brain connectivity was especially strong at frontal and central regions, during both solo and joint playing.

*Statistical evaluation of graph-theoretical measures for ACI and PSI*

*Degrees and Strengths*

Strengths averaged across participants separately for the three play conditions and 21 electrode sites are represented in Supplementary Figure S5 for some frequencies of interest (6, 10, 16 and 28 Hz). At higher frequencies (8 Hz and higher), strengths (for both *PSI* and *ACI*) of the playing guitarists within their brains were higher than that of listening guitarists. In addition, there were lower strengths between the brains in the duo-play condition in the alpha frequency range, especially at 10 Hz, and high strengths in the beta1 frequency range, especially at 16 Hz.

For statistical analyses, individual frequencies were collapsed into five bands: delta (2-3 Hz), theta (4-7 Hz), alpha (8-12 Hz), beta1 (14-20 Hz) and beta2 (24-28 Hz), and individual electrodes were collapsed into three regions along the anterior-posterior axis (frontal, central and parieto-occipital). The strengths were determined for the whole network of the duet, encompassing both within- and between-brain connections, as well as separately for within- and between-brain networks. For all the networks, four-way repeated-measures ANOVAs (Guitarist x Play Condition x Frequency Band x Site) were carried out. Mean and standard deviation for strengths based on *ACI* and *PSI* measures are presented in Table S1, calculated separately for intra- and inter-brain connections in the five frequency bands. Corresponding ANOVA results (significant effects only) for hyper-, intra-, and inter-brain connections are presented in Table S2. For the *ACI*, between-brain connections decreased, and within-brains connections increased with higher frequency at parieto-occipital sites only. In the case of the *PSI*, strengths decreased with higher frequency for both intra- and inter-brain connections.

*Clustering coefficient (CC) and characteristic path length (CPL)*

*CC* and *CPL* were determined across all pairs of guitarists for different frequency bins and play conditions, taking into account intra- and inter-brain connections. Particular frequencies were then collapsed into the five frequency bands and analyzed using two-way repeated measures ANOVA (Play Condition x Frequency Band).

Both measures (*ACI* and *PSI*) showed a significant main effect of Frequency Band only, indicating significant changes across the frequency bands: CC increased with higher frequencies for the ACI (F(4,28) = 11.5, P<0.0001,  = 0.62, but decreased for the PSI, F(4,28) = 149.3, P<0.0001,  = 0.96; CPL increased with higher frequencies for both the ACI, F(4,28) = 52.4, P<0.0001,  = 0.88) and especially the PSI, F(4,28) = 757.9, P<0.0001,  = 0.99, measure.

*Small-worldness of hyper-brain networks*

The small-world characteristics (*σ* and *ω*) in the different frequency bands averaged across the three playing conditions and all guitarist pairs are presented in Figure S6. According to the small-world coefficient *σ*, the hyper-brain networks correspond to SWN only for the *ACI* measures, wherein *σ* for *ACI* decreases with higher frequency. The small-world coefficient *σ* for the networks based on *PSI* measure is smaller than 1 for all frequency bands and decreases from delta to beta. The small-world coefficient *ω* decreases with higher frequency for both measures and ranges between -1.04 and -0.67 for the *ACI*, and between -0.71 and -0.34 for the *PSI* measure. This indicates that networks based on *ACI* and *PSI* are (in contrast to *ICI*) very regular networks.

*Modularity (M), community structures and the Z-P parameter space*

Modularity (*M*), which was analyzed in the same way as *CC* and *CPL,* showed a significant increase with frequency for both measures, *ACI*: F(4,28) = 40.5, P<0.0001,  = 0.85; *PSI*: F(4,28) = 201.8, P<0.0001,  = 0.97, pointing towards a stronger partitioning of networks synchronizing or communicating at high frequencies (e.g., beta frequency bands).

To define how nodes were positioned in their own module and with respect to other modules, we calculated the within-module degree (*Z_i_*) and participation coefficient (*P_i_*) of the node i for the whole network of a given pair (connections within and between the brains). Within-module degree measures how ‘well-connected’ node i is to other nodes in the module, whereas the participation coefficient reflects how ‘well-distributed’ the links of the node i are among the other modules. Together, *Z_i_* and *P_i_* form the so-called Z-P parameter space, whose partitions indicate specific node roles.

Depending on the within-module degree (*Z_i_*) and participation coefficient (*P_i_*), we divided the nodes in the Z-P parameter space into eight different roles: (R1) ultra-peripheral non-hubs (P ≤ 0.05); (R2) peripheral non-hubs (0.05< P ≤ 0.5); (R3) connector non-hubs (0.5 < P ≤ 0.8); (R4) kinless non-hubs (0.8 < P ≤ 1.0); R5-R8 are then ultra-peripheral, peripheral, connector, and kinless hubs, correspondingly. We calculated the number of nodes falling into these regions. Results of this calculation at different frequencies are displayed in Figures S7 and S8 for the *ACI* and the *PSI*, respectively. It can be seen that the number of connector nodes for both *ACI* and the *PSI* decreased with higher frequency, whereas the number of ultra-peripheral nodes decreased for *PSI* but increased for *ACI* and the number of peripheral nodes, increased for *PSI* but decreased for *ACI*. These tendencies apply to both hubs and non-hubs, at least for *ACI*. Interestingly, there was a higher number of non-hub connectors during separate playing (Play A and Play B) than during joint playing (Play AB) at the alpha frequency (8-12 Hz), whereas the joint playing was accompanied by a higher number of hub-connectors at the delta (2-3 Hz) and theta (5-7 Hz) frequency. We note that the present observations of differences between joint and separate playing are not backed up by inference statistics, and hence should be treated with caution.

In summary, the described coupling indices (*ACI* and *PSI* together with *ICI*) measure different aspects of phase synchronization and are suitable for single-trial analysis: *ACI* describes *in-phase* synchronization, whereas *PSI* reflects undirected coupling regardless of the phase angle. *ICI* is derived from absolute and positive changes in phase differences (see Methods), and indicates both the strength of the in-phase coupling and the phase earliness of one time series relative to the other. Thus, these three indices reflect different neural coupling dynamics and are rather complementary to each other.
